# Transcriptomic, proteomic, and metabolomic analyses identify candidate pathways linking maternal cadmium exposure to altered neurodevelopment and behavior

**DOI:** 10.1038/s41598-021-95630-2

**Published:** 2021-08-11

**Authors:** Kathleen M. Hudson, Emily Shiver, Jianshi Yu, Sanya Mehta, Dereje D. Jima, Maureen A. Kane, Heather B. Patisaul, Michael Cowley

**Affiliations:** 1 Department of Biological Sciences, North Carolina State University, Raleigh, NC 27695 USA; 2 Center for Human Health and the Environment, North Carolina State University, Raleigh, NC 27695 USA; 3 School of Pharmacy Mass Spectrometry Center, Department of Pharmaceutical Sciences, University of Maryland, Baltimore, MD 21201 USA; 4 Bioinformatics Research Center, North Carolina State University, Raleigh, NC 27695 USA

**Keywords:** Developmental biology, Proteomics, Transcriptomics, Development of the nervous system, Myelin biology and repair, Social behaviour

## Abstract

Cadmium (Cd) is a ubiquitous toxic heavy metal of major public concern. Despite inefficient placental transfer, maternal Cd exposure impairs fetal growth and development. Increasing evidence from animal models and humans suggests maternal Cd exposure negatively impacts neurodevelopment; however, the underlying molecular mechanisms are unclear. To address this, we utilized multiple -omics approaches in a mouse model of maternal Cd exposure to identify pathways altered in the developing brain. Offspring maternally exposed to Cd presented with enlarged brains proportional to body weights at birth and altered behavior at adulthood. RNA-seq in newborn brains identified exposure-associated increases in *Hox* gene and myelin marker expression and suggested perturbed retinoic acid (RA) signaling. Proteomic analysis showed altered levels of proteins involved in cellular energy pathways, hypoxic response, and RA signaling. Consistent with transcriptomic and proteomic analyses, we identified increased levels of retinoids in maternally-exposed newborn brains. Metabolomic analyses identified metabolites with significantly altered abundance, supportive of changes to cellular energy pathways and hypoxia. Finally, maternal Cd exposure reduced mitochondrial DNA levels in newborn brains. The identification of multiple pathways perturbed in the developing brain provides a basis for future studies determining the mechanistic links between maternal Cd exposure and altered neurodevelopment and behavior.

## Introduction

Exposure to heavy metals during pregnancy can impact fetal development and have lasting consequences to the health of the offspring. In utero exposure has been associated with impaired growth, metabolic dysfunction, altered neurodevelopment and behavior, and cancer^1,2^. Of the ten chemicals of major public health concern identified by the World Health Organization, four are heavy metals: arsenic (As), lead (Pb), mercury (Hg), and cadmium (Cd)^3^.

Cd is found naturally at low concentrations in the soil, but levels of human exposure have dramatically increased since the early twentieth century due to anthropogenic activities such as fossil fuel combustion and metal mining and refining^4^. Inhalation and ingestion are the primary routes of Cd exposure in humans. Cigarette smoke, contaminated dust, and occupational exposure are the primary sources of inhaled Cd. Among non-smokers, Cd ingestion of contaminated produce or water is the primary route of exposure^5,6^. Exposure to high levels of Cd during adulthood is associated with cancer, hepatotoxicity, renal toxicity, and softening of the bones^4,7,8^; however, it is becoming clear that chronic exposure to low levels of Cd is associated with noncommunicable metabolic diseases such as obesity, type II diabetes, non-alcoholic fatty liver disease, and cardiovascular disease^9–12^.

There is also mounting evidence that maternal exposure to Cd negatively affects fetal health^13^. While some heavy metals such as Pb readily cross the placenta^14^, Cd accumulates in the placenta and does not efficiently enter fetal circulation^1^. Despite this, maternal Cd exposure has been associated with altered trace metal homeostasis^15^, reduced fetal growth^16^, congenital malformations^17^, increased heart weight at birth, susceptibility to hypertension during adulthood^15^, and impaired neurodevelopment^18^.

Human epidemiological studies have linked higher levels of Cd in cord or maternal blood with lowered intelligence, altered motor development, and behavioral deficits in children^18–20^; these findings have been supported by animal models. Rats exposed to Cd through their mothers’ milk exhibited increased motor activity during adulthood^21^. An additional study examined the behavior of rat pups born to mothers who were injected with Cd during the organogenesis period (days 7–15 of gestation) and found delayed sensorimotor development and reduced anxiety during adulthood^22^. Furthermore, a recent study in zebrafish found that parental exposure to Cd prior to mating had neurotoxic effects on the F_1_ offspring^23^.

Despite strong evidence that parental and developmental Cd exposure affects neurodevelopment and behavior, the impact of maternal Cd exposure on molecular processes in the developing brain are not as well studied. Neonatal and pubertal rat pups born to mothers who were exposed to Cd during pregnancy and lactation had increased brain weights relative to body weight, altered brain lipid peroxidation, and severely damaged mitochondria in the brain^24^. Mice that were maternally exposed to Cd from gestational day 1 to postnatal day 10 showed perturbed expression of sex hormone receptor genes in the brain^25^ and altered thyroid hormone metabolism—an essential process in brain development^26^. While these targeted studies describe specific pathways modulated by maternal Cd exposure, whether or not these represent the full extent of molecular changes in the developing brain is unclear. Global transcriptomic, proteomic, and metabolomic analyses of the developing brain could identify novel pathways and mechanisms that underpin Cd-associated neurodevelopmental and behavioral changes.

Therefore, we established a mouse model of maternal Cd exposure in which F_0_ females are exposed to a low, environmentally relevant dose or a high dose of Cd through their drinking water for 5 weeks prior to gestation until parturition. We utilized whole transcriptome, proteome, and metabolome approaches to identify molecular changes in newborn F_1_ brains following maternal Cd exposure. Through these -omic approaches, we identify multiple altered pathways and provide a foundation for future mechanistic studies linking developmental Cd exposure with altered neurodevelopment and behavior.

## Results

### Maternal Cd exposure increases proportional brain weight at birth

The exposure model and hybrid mating strategy utilized in this study are described in detail in previous work^15^. Briefly, F_0_ mothers were administered one of three Cd doses chronically through their drinking water before and during pregnancy: 0 ppm (control), 1 ppm (low, environmentally relevant dose), and 50 ppm (high dose consistent with other rodent studies). For ease of annotation, the generation of the mice and the Cd dose to which they were exposed will be herein referred to as: F_(generation)_^(Cd dose)^. For example, F_0_^1ppm^ refers to mothers exposed to 1 ppm Cd and F_1_^50ppm^ refers to offspring maternally exposed to 50 ppm Cd. A hybrid mating scheme between C57BL/6J (‘B’) and CAST/EiJ (‘C’) inbred mouse strains was utilized to analyze parent-of-origin effects in a separate analysis (Hudson and Cowley, unpublished). Herein, F_1_ mice will be referred to by their maternal × paternal cross, i.e. B × C (B mother × C father) or C × B (C mother × B father). F_1_ mice were dissected within 24 h of birth and herein will be referred to as ‘newborn’.

**Figure 1:**
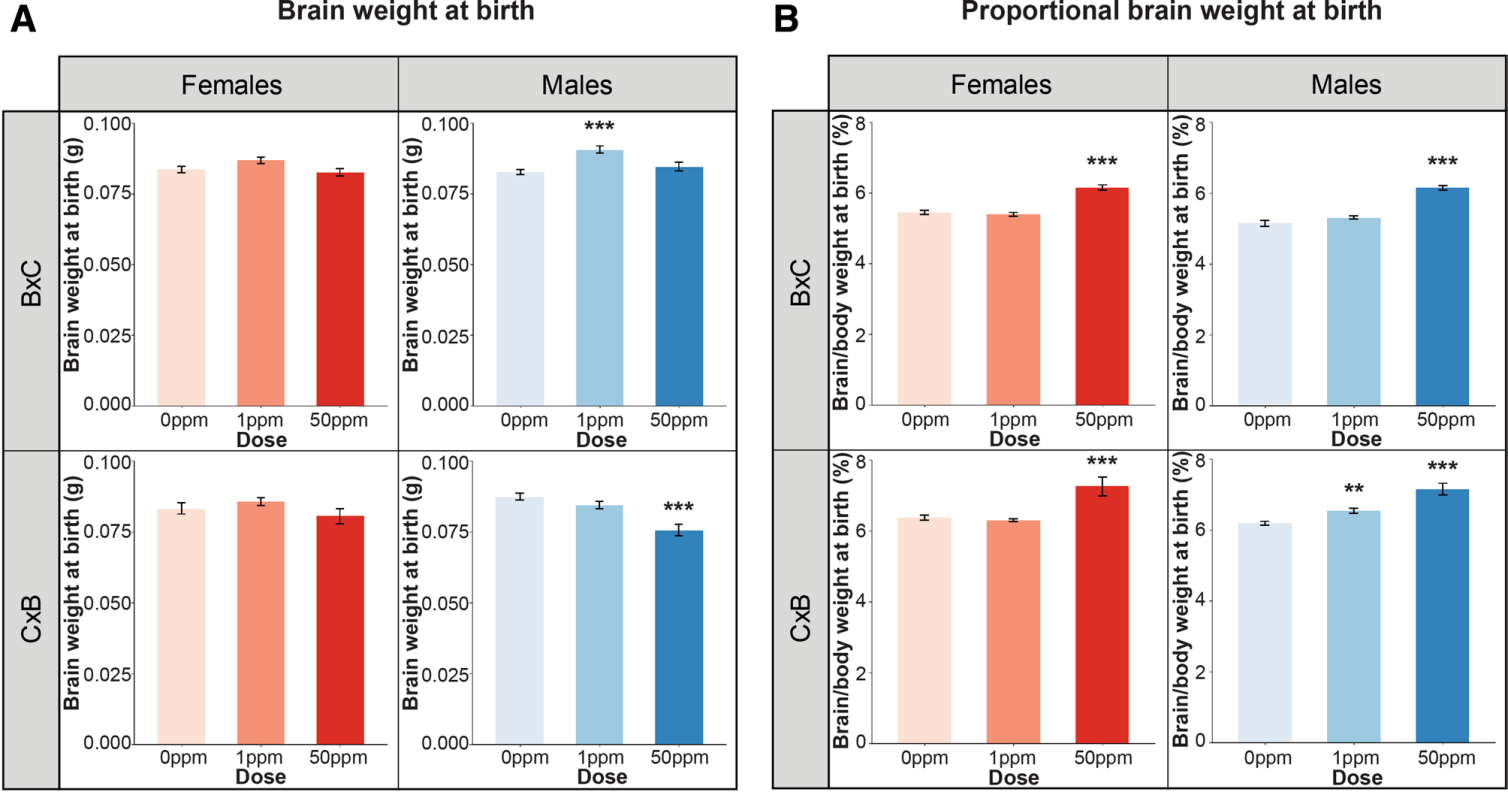
The effects of maternal Cd exposure on raw brain weight and normalized brain weight at birth. (**A**) Raw brain weight at birth. (**B**) Proportional brain weight at birth. *p < 0.05, **p < 0.01, ***p < 0.001 (one-way ANOVA with post-hoc Dunnett's test comparing 1 ppm and 50 ppm to 0 ppm).

The only statistically significant differences observed in raw brain weights at birth following maternal Cd exposure were in B × C F_1_^1ppm^ males (increase) and C × B F_1_^50ppm^ males (decrease); the remaining groups showed no significant differences (Fig. 1A). We previously showed that maternal Cd exposure leads to a significant reduction in body weight at birth^15^. When brain weight was normalized to body weight to account for these differences, a significant increase in the proportional weight of the brain was observed at birth in the F_1_^50ppm^ pups of both crosses and both sexes, as well as the F_1_^1ppm^ C × B males (Fig. 1B). Differences in raw or proportional brain weights were not observed at 6 months, with the exception of a significant increase in proportional brain weight that persisted in C × B F_1_^50ppm^ females (Supplementary Fig. 1).

To identify any significant cell damage due to maternal Cd exposure, hematoxylin and eosin staining was performed on serial coronal sections of whole newborn F_1_^0ppm^ and F_1_^50ppm^ B × C male and female brains. No overt differences in cell damage or death were identified in corresponding regions between individuals of different maternal Cd doses (data not shown).

### Maternal Cd exposure programs perturbed anxiety-related behavior during adulthood

Due to the significant increase in proportional brain weight at birth and the previously reported association of early life exposure to Cd with altered behavior, Social Interaction (SI) and Open Field (OF) behavioral tests were performed in aged F_1_ mice 2–4 weeks prior to sacrifice (6 months) to test whether altered behavior in adulthood can be programmed by maternal Cd exposure alone. The SI test is used to assess sociability in the presence of a novel animal^27^, while the OF test is used to measure activity, anxiety, and willingness to explore^28^.

**Figure 2:**
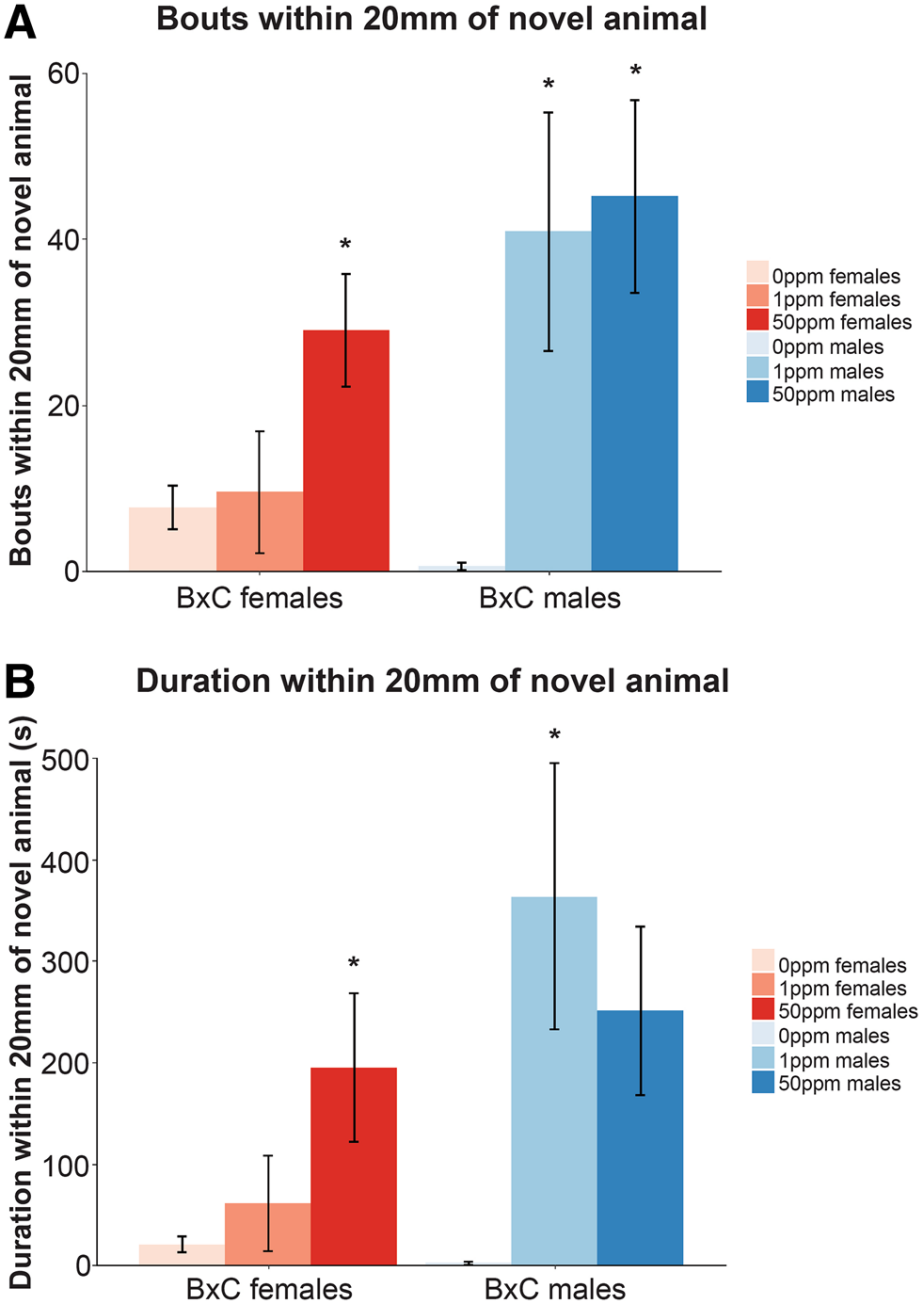
Select SI behavioral measurements in B × C mice maternally exposed to Cd. (**A**) Bouts within 20 mm of novel animal. (**B**) Duration within 20 mm of noval animal. *p < 0.05, **p < 0.01, ***p < 0.001 (one-way ANOVA with post-hoc Dunnett's test comparing 1 ppm and 50 ppm to 0 ppm).

Maternal Cd exposure had a more profound and diverse effect on sociability behaviors assessed in the SI test compared to the anxiety-related behaviors assessed in the OF test. F_1_^50ppm^ B × C females and both exposure groups of B × C F_1_ males investigated the novel animal more frequently than control mice, and F_1_^50ppm^ B × C females and F_1_^1ppm^ B × C males spent a longer duration of time near the novel animal than control mice (Fig. 2, Supplementary Table 1). Maternally-exposed B × C animals appeared to initially investigate the novel animal more quickly than controls, though this difference was not statistically significant (Supplementary Table 1). In contrast to B × C animals, maternally-exposed C × B animals appeared to investigate the novel animal less frequently and took longer to initially investigate than controls, though these data were not statistically significant (Supplementary Table 1).

In the OF testing, C × B F_1_^1ppm^ males showed a significant increase in activity (as indicated by an increased number of visits to both the perimeter and center), willingness to explore the center of the box, and took less time to initially explore the center compared to control C × B males (Supplementary Table 1). This was a similar, yet non-significant trend in other maternally-exposed mice (Supplementary Table 1). B × C F_1_^1ppm^ females visited the perimeter of the box significantly more than control B × C females, indicative of more anxious behavior (Supplementary Table 1). These behavioral data indicate that, despite the consistent effect of maternal Cd exposure on newborn brain weight, the effect of exposure on offspring behavior at adulthood may vary depending on genetic background. This might be in part due to some significant differences in baseline behaviors between the four control groups (Supplementary Table 1).

Due to the robust Cd-associated behavioral changes observed in B × C F_1_ females, as well as our previously published work that identified Cd-associated transcriptomic changes in the newborn heart—which would therefore permit us to identify conserved gene expression changes in multiple organs due to maternal Cd exposure, these animals were selected for further molecular analyses. Subsequent data presented here were obtained in these animals unless otherwise specified.

### Transcriptomic analysis of newborn brains identifies maternal Cd-associated activation of myelin markers

**Figure 3:**
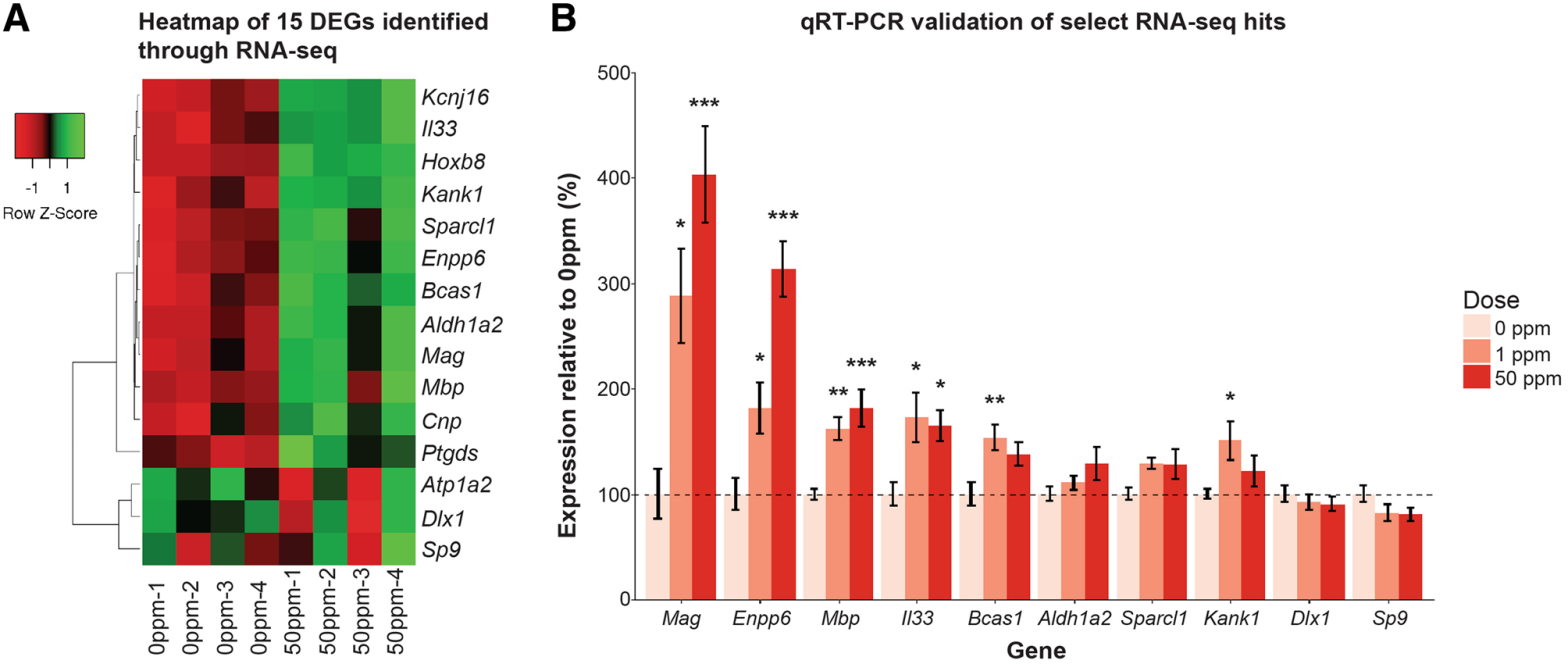
RNA-seq heatmap and qRT-PCR validations. (**A**) Heatmap of the 15 significantly differentially expressed genes in newborn B × C female whole brains as a results of 50 ppm maternal Cd exposure. n=4 for 0 ppm and 50 ppm groups. (**B**) qRT-PCR validation of select RNA-seq hits in newborn B × C female brains due to maternal Cd exposure. n=8 for each dose. *p < 0.05, **p < 0.01, ***p < 0.001 (one-way ANOVA with past-hoc Dunnett's test comparing 1 ppm and 50 ppm to 0 ppm).

In order to understand how maternal Cd exposure alters pathways that could contribute to enlarged proportional brain weight at birth and perturbed behavior at adulthood, RNA-seq was performed on whole brains of newborn F_1_^0ppm^ and F_1_^50ppm^ B × C females (n = 4 for each dose with each individual birthed in a different litter). After quality control filtering, over 13,000 genes mapped to both parental genomes (14,417 aligned to B genome, 13,277 aligned to C genome, Supplementary Table 2). From these, 15 differentially expressed genes (DEGs) that were significant after multiple testing correction and common to both parental genome alignments were identified (Table 1, Fig. 3A). Select DEGs were validated with qRT-PCR, including on F_1_^1ppm^ B × C females (n = 8 for each dose, Fig. 3B).

**Table 1:** List of 15 significantly differentially expressed genes in newborn B × C female brains as a result of 50 ppm maternal Cd exposure and their relevance to myelin. Numerical values are derived from the alignment to the B genome.

Gene	Log2Fold Change	Adjusted p-value	Associated with oligodendrocytes or myelination	Part of myelin transcriptome^30^
*Hoxb8*	6.87	6.71E−14		
*Bcas1*	0.98	2.26E−05	X^30,33^	X
*Il33*	0.89	3.02E−03	X^30,34^	X
*Enpp6*	1.66	3.02E−03	X^35^	
*Mag*	1.16	8.21E−03	X^30,36^	X
*Kank1*	0.48	8.88E−03	X^37^	
*Sparcl1*	0.43	8.88E−03		
*Aldh1a2*	0.44	8.88E−03	X^30,38^	X
*Sp9*	−0.40	8.88E−03	X^30^	X
*Dlx1*	−0.42	1.24E−02	X^39^	
*Atp1a2*	0.29	1.47E−02		
*Mbp*	0.49	3.13E−02	X^30,40^	X
*Kcnj16*	0.69	3.75E−02	X^41^	
*Cnp*	0.52	4.16E−02	X^30,42^	X
*Ptgds*	0.51	4.88E−02	X^30,43^	X

Twelve of the fifteen DEGs are associated with oligodendrocytes or myelination (Table 1), a process performed by oligodendrocytes after birth in the mouse^29^. Eight of the fifteen DEGs are part of the myelin transcriptome^30^, and seven of these eight DEGs had increased expression due to maternal Cd exposure (Table 1). Term enrichment analysis for the 15 DEGs showed a significant enrichment for terms related to retinoic acid (RA) signaling (Supplementary Table 2, terms with adj p val < 0.05 highlighted in red), which is required prior to the onset of myelination^31^. Based on these observations, we hypothesized that the process of myelination is occurring earlier in maternally-exposed F_1_ mice. However, we were unable to detect myelin in the prefrontal cortex of these animals at birth using histological approaches (Black-Gold II stain, data not shown), consistent with previous work showing that myelin is challenging to visualize in rodents during early postnatal life due to its low abundance and despite the presence of molecular markers of myelin^29,32^.

### Maternal Cd exposure perturbs *Hox* gene expression in the newborn brain

**Figure 4:**
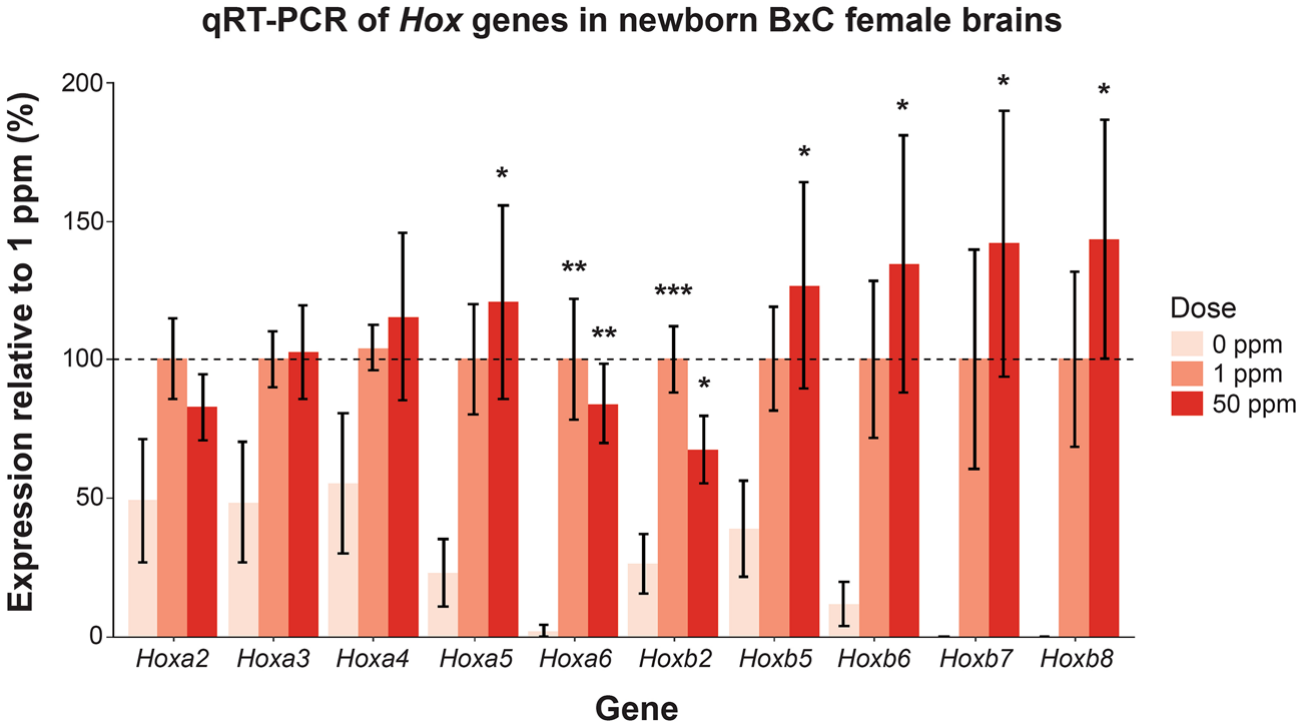
qRT-PCR of Hox genes in newborn B × C female brains exposed maternally to Cd. Individuals with undetectable Ct values were assigned expression values of 0%. Expression data shown relative to mean of 1 ppm (normalized to 100%) for each gene due to several genes having undetectable levels in controls. *p < 0.05, **p < 0.01, ***p < 0.001 (one-way ANOVA with post-hoc Dunnett's test comparing 1 ppm and 50 ppm to 0 ppm).

RNA-seq identified *Hoxb8* as the most significantly differentially expressed gene in the brain due to maternal Cd exposure (Table 1). *Hoxb8* demonstrated negligible expression in control brains, consistent with a more posterior expression pattern during normal development^44^, yet was significantly increased in the brains of mice maternally-exposed to Cd. Further evaluation of the RNA-seq data suggested that other *Hox* genes may be similarly affected, although their differences in expression did not achieve statistical significance using this approach (Supplementary Table 2). To further assess the extent of *Hox* gene misregulation by maternal Cd exposure, the expression of all *Hox* 1–9 genes contained in the A-D clusters was quantified with qRT-PCR in newborn B × C female brains exposed maternally to 0 ppm, 1 ppm, or 50 ppm (n = 8 for each group). Consistent detectable amplification (see Materials and Methods for criteria) in at least one of the three treatment groups was only found in genes in the *Hoxa* and *Hoxb* clusters (Fig. 4). Expression data were normalized to the 1 ppm group in each respective gene due to undetectable amplification of several *Hox* genes in multiple control samples (see Materials and Methods for detailed explanation). 50 ppm maternal Cd exposure significantly increased the expression of *Hoxb2* and more posteriorly expressed *Hoxa* and *Hoxb* genes (i.e. *Hoxa5* through *Hoxa6* and *Hoxb5* through *Hoxb8*) when compared to controls, and 1 ppm maternal Cd exposure significantly increased the expression of *Hoxa6* and *Hoxb2* when compared to controls (Fig. 4).

### Proteomic analysis of newborn brains identifies maternal Cd-associated changes in the abundance of proteins related to cellular energy, hypoxia, histone 1 subunits, myelin, and retinoic acid

To determine whether the observed changes in gene expression correlated with altered abundances at the protein level, a discovery-based mass spectrometry proteomics approach was performed on newborn B × C female whole brains. 46 proteins were identified as significantly different in abundance in the F_1_^50ppm^ brains compared to controls after correcting for multiple testing and filtering hits based on criteria described in the Materials and Methods (Table 2, raw data in Supplementary Table 3). In general, a dose-dependent increase or decrease in protein abundance was seen as a result of maternal Cd exposure; however, this was only significant for 6/46 proteins in the F_1_^1ppm^ brains when compared to controls.

**Table 2:** Significantly differentially abundant proteins in the F_1_^50ppm^ B × C female newborn brains compared to controls, ranked by adj p val in 50 ppm group. Abundance ratios for these proteins in the F_1_^1ppm^ female newborn brains included. ^a^ = found in myelin proteome, ^b^ = involved in RA pathways, ^c^ = histone 1 subunit.

Protein	Abundance Ratio Adj. P-Value: (1 ppm)/(0 ppm)	Abundance Ratio Adj. P-Value: (50 ppm)/(0 ppm)	Abundances (Grouped): 0 ppm	Abundances (Grouped): 1 ppm	Abundances (Grouped): 50 ppm	1 ppm change to 0 ppm (%)	50 ppm change to 0 ppm (%)
Ldha^a,b^	0.973898894	0.000004728	90.9	91.8	117.3	0.990099	29.042904
Pfkl	0.119323304	0.000004768	86.1	98.2	115.7	14.053426	34.37863
Slc6a11	0.046109095	0.000011345	88.7	97.7	113.6	10.146561	28.072153
Nefm^a,b^	0.000014534	0.000013915	84.7	104.7	110.6	23.612751	30.578512
Gpi1^a^	0.99117914	0.00012503	90.7	92.5	116.9	1.9845645	28.886439
Aldoc^a,b^	0.014283746	0.0002136	86.9	102.2	110.8	17.606444	27.502877
Hist1h1e^c^	0.680950614	0.000458262	108.9	103.4	87.7	−5.050505	−19.4674
Slc1a3^a,b^	0.026706171	0.000459068	94.3	95	110.7	0.7423118	17.391304
Srsf4^b^	0.254416432	0.000500359	109.8	102	88.2	−7.103825	−19.67213
Tuba4a^a,b^	0.812211612	0.001034191	91	100.3	108.7	10.21978	19.450549
Hpca	0.818093421	0.001676617	92	96.1	111.9	4.4565217	21.630435
Tpi1^a,b^	0.995738881	0.001737399	94.3	93.7	111.9	−0.636267	18.663839
Ahsg^b^	0.259432391	0.001766066	98.4	86.8	114.8	−11.78862	16.666667
Fxyd6	0.084419728	0.0037873	91.9	98	110.1	6.6376496	19.804135
Ckmt1	0.991151472	0.004356406	94.8	94.8	110.3	0	16.350211
Hist1h1b^c^	0.995738881	0.004617561	105.6	108.1	86.2	2.3674242	−18.37121
Hprt	0.926735568	0.004685861	95.2	89.9	114.9	−5.567227	20.693277
Aldoa^a,b^	0.937783837	0.004964389	92.4	93.8	113.9	1.5151515	23.268398
Hist1h1d^b,c^	0.606980956	0.004964389	108.6	102.1	89.2	−5.985267	−17.86372
Ptma^b^	0.080791892	0.004964389	112.2	97.6	90.2	−13.01248	−19.60784
Glul^a,b^	0.729109325	0.005102008	91.5	98.8	109.8	7.9781421	20
Prkacb^b^	0.063552234	0.005317616	99.5	90.1	110.4	−9.447236	10.954774
Sncb^a^	0.585421643	0.005422114	93.9	91	115.1	−3.088392	22.57721
Slc12a5	0.970511751	0.006450782	91.6	95.3	113.1	4.0393013	23.471616
Apoe^b^	0.680950614	0.008266455	89.6	96.6	113.7	7.8125	26.897321
Alg2	0.31039933	0.008520425	90.4	98	111.6	8.4070796	23.451327
Apoa1	0.991151472	0.009864999	89.7	94.9	115.4	5.7971014	28.651059
Eno1^a,b^	0.674411211	0.010566996	93.7	92.4	113.9	−1.387407	21.558164
Abat^b^	0.989354576	0.012954966	92.4	94	113.6	1.7316017	22.943723
Crabp1^b^	0.04344094	0.013498454	85.5	103.8	110.7	21.403509	29.473684
Camk2d^a,b^	0.580890155	0.018126666	90.3	96.7	113	7.0874862	25.138427
Satb2	0.973898894	0.01885168	108.5	110.1	81.4	1.4746544	−24.97696
Ndrg2^b^	0.680375748	0.018905298	94.6	95.9	109.5	1.3742072	15.750529
Dnm1^a,b^	0.174291855	0.022293486	93.1	98.7	108.2	6.0150376	16.219119
Trf	0.176950766	0.025109111	96.1	91.7	112.2	−4.578564	16.753382
Vsnl1^a,b^	0.909390199	0.029396495	97.2	93.2	109.6	−4.115226	12.757202
Gstm1^a,b^	0.989354576	0.031417367	93.9	93	113.1	−0.958466	20.447284
Set	0.031217009	0.031978606	106.2	99	94.8	−6.779661	−10.73446
Syn2^a^	0.426588152	0.032509188	88.8	100.9	110.3	13.626126	24.211712
Anxa2^a,b^	0.797814818	0.03380452	95.3	95.6	109.1	0.3147954	14.480588
Trp53i11	0.737170178	0.034474205	107.9	103	89.2	−4.541242	−17.33086
Hpcal1^a,b^	0.818093421	0.038283218	94.2	96.9	108.9	2.866242	15.605096
Stmn2	0.419569789	0.039318421	89.2	102.9	107.9	15.358744	20.964126
Camk2g^a,b^	0.836358682	0.043020031	87.7	102.9	109.3	17.331813	24.629418
Dpp6^a,b^	0.389249664	0.04777657	88.9	97.4	113.7	9.5613048	27.896513
Glud1^b^	0.474755049	0.048280644	92.8	98.4	108.9	6.0344828	17.349138

Among the 46 differentially abundant proteins, there was a significant enrichment for terms related to cellular energy and metabolism pathways and hypoxia (Supplementary Table 4), consistent with a maternal Cd exposure-induced Fe deficiency that we reported previously^15^. Additionally, three histone 1 subunits were significantly decreased in F_1_^50ppm^ brains compared to controls (Table 2). Consistent with findings from RNA-seq, 20 of the 46 differentially abundant proteins in the F_1_^50ppm^ brains are known to be part of the myelin proteome^45^ (Supplementary Table 4), and all twenty were significantly increased in abundance (Table 2). The most significant term in the ESCAPE enrichment analysis predicted an increase in *Sox2* (Supplementary Table 4), which is essential for the proliferation and differentiation of oligodendrocytes during postnatal myelination^37^. Consistent with the findings of RNA-seq, there was a significant enrichment for terms related to RA (Supplementary Table 4).

### Maternal Cd exposure leads to reduced mitochondrial content in the brain at birth

**Figure 5:**
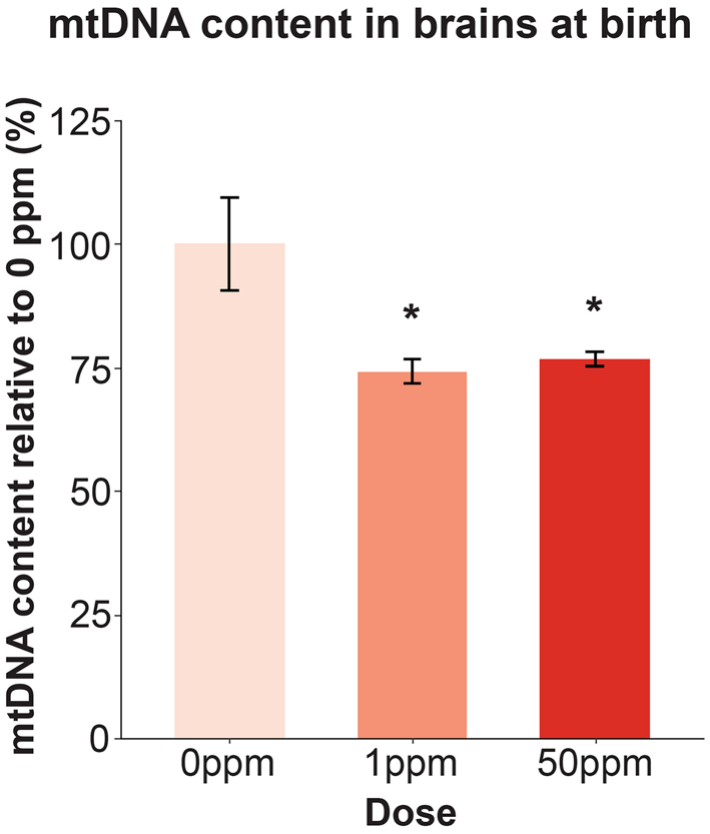
mtDNA content in B × C female newborn brains as a result of maternal Cd exposure. mtDNA content quantified through qPCR. *p < 0.05, **p < 0.01, ***p < 0.001 (one-way ANOVA with post-hoc Dunnett's test comparing 1 ppm and 50 ppm to 0 ppm).

Previous work by another group found severely damaged mitochondria in electron micrographs of brains from rats whose mothers were exposed to Cd during pregnancy and lactation^24^. Given this and our proteomic analysis showing alterations in proteins involved in cellular energy pathways, we quantified mitochondrial DNA (mtDNA) content in newborn B × C female brains. mtDNA content, as quantified through qPCR, was significantly reduced by approximately 25% in newborn B × C female brains as a result of both 1 ppm and 50 ppm maternal Cd exposure (Fig. 5).

### Metabolomic analyses of newborn brains in response to maternal Cd exposure

We previously showed maternal Cd exposure leads to a significant iron deficiency in newborn pups^15^. Considering the iron deficiency and the proteomic data containing a significant enrichment for terms related to hypoxia and cellular energy and metabolism pathways, we hypothesized that the iron deficiency was leading to a hypoxic environment, resulting in an increase in anaerobic respiration in the brain to meet glucose demands. We therefore quantified the metabolite lactate, a major byproduct of anaerobic respiration that increases in hypoxic conditions^46^ in a small subset of B × C female newborn brains. There were no statistically significant differences in lactate quantified as mM per mg of dry extract weight between the three treatment groups, potentially due to our limited sample size (Supplementary Fig. 2A). We hypothesized that myelination may be occurring earlier in neurodevelopment due to maternal Cd exposure, which may alter the lipid proportional weight in the brain. To account for differences in composition of extract weights sampled for each individual as a proportion of their total brain weight, lactate data were normalized to proportion of brain weight. These data were more suggestive of a dose-dependent increase in lactate, though these data were not statistically significant (Supplementary Fig. 2B).

**Figure 6:**
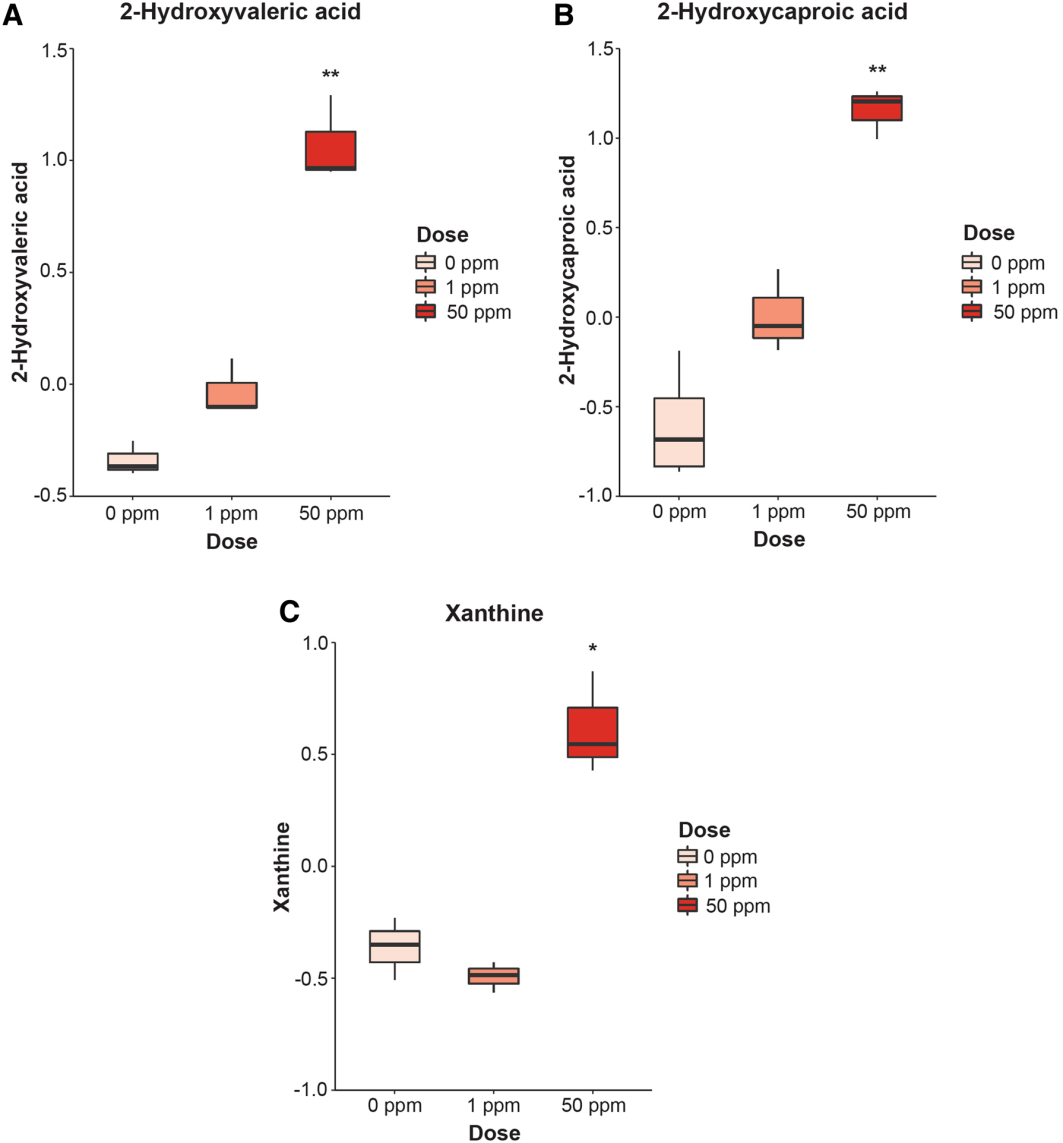
Metabolites identified as significantly different in metabolomics data. (**A**) 2-Hydroxyvaleric acid. (**B**) 2-Hydroxycaproic acid. (**C**) Xanthine. The y-axis units for all graphs are normalized peak area as calculated in MetaboAnalyst software. *p < 0.05, **p < 0.01, ***p < 0.001 (one-way ANOVA with post-hoc Dunnett's text comparing 1 ppm and 50 ppm to 0 ppm, followed by Benjamini-Hochberg correction for multiple testing).

We then performed a mass spectrometry discovery-based approach for identifying metabolites that were differentially abundant in F_1_^1ppm^ and F_1_^50ppm^ B × C female newborn brains when compared to F_1_^0ppm^ controls. Five metabolites were identified in the F_1_^1ppm^ B × C females, while 14 metabolites were identified in the F_1_^50ppm^ B × C females (Supplementary Table 5). However, after multiple testing correction, only 3 metabolites remained significantly different in the F_1_^50ppm^ B × C females: 2-hydroxyvaleric acid, 2-hydroxycaproic acid, and xanthine (Fig. 6 metabolomics, Supplementary Table 5).

### Maternal Cd exposure leads to increased levels of retinoic acid in the brain at birth

**Figure 7:**
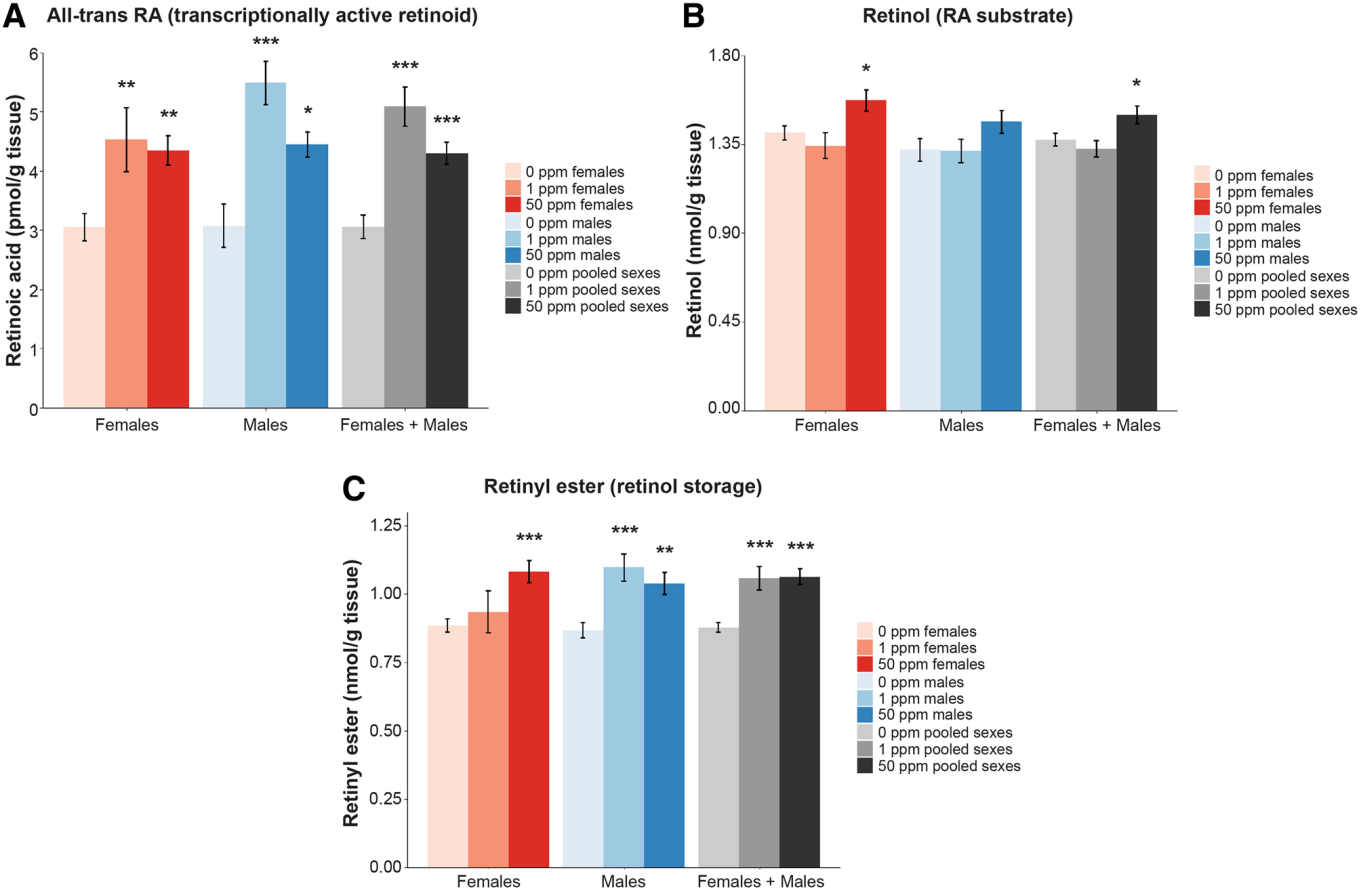
Mass-spec quantification of retinoid species in newborn B × C brains as a result of maternal Cd exposure. (**A**) All-trans RA. (**B**) Retinol. (**C**) Retinyl ester. *p < 0.05, **p < 0.01, ***p < 0.001 (one-way ANOVA with post-hoc Dunnett's test comparing 1 ppm and 50 ppm to 0 ppm).

Given our observations of altered levels of RA-associated transcripts and proteins in brains of maternal Cd-exposed mice, and the known role of RA in behavior^47^, myelination^31^, and regulation of *Hox* gene expression^44^, we hypothesized that RA levels in the brain were perturbed at birth as a result of maternal Cd exposure. To test this, we quantified three retinoid species in newborn B × C male and female whole brains: all-trans RA, retinol (RA substrate), and retinyl ester (retinol storage). Maternal Cd exposure led to significantly increased levels of RA in the brain of both F_1_^1ppm^ and F_1_^50ppm^ pups (Fig. 7A). The F_1_^1ppm^ pups tended to have higher brain levels of RA than the F_1_^50ppm^ pups, though this was not statistically significant. Retinol levels were significantly increased as a result of 50 ppm maternal Cd exposure in females or when the data from both sexes were pooled (Fig. 7B). Retinyl ester was significantly increased due to 1 and 50 ppm maternal Cd doses in males and when the data for sexes were pooled, but was only significantly increased in F_1_^50ppm^ female brains (Fig. 7C). Taken together, these data suggest increased biosynthesis and/or reduced degradation of RA.

## Discussion

**Figure 8:**
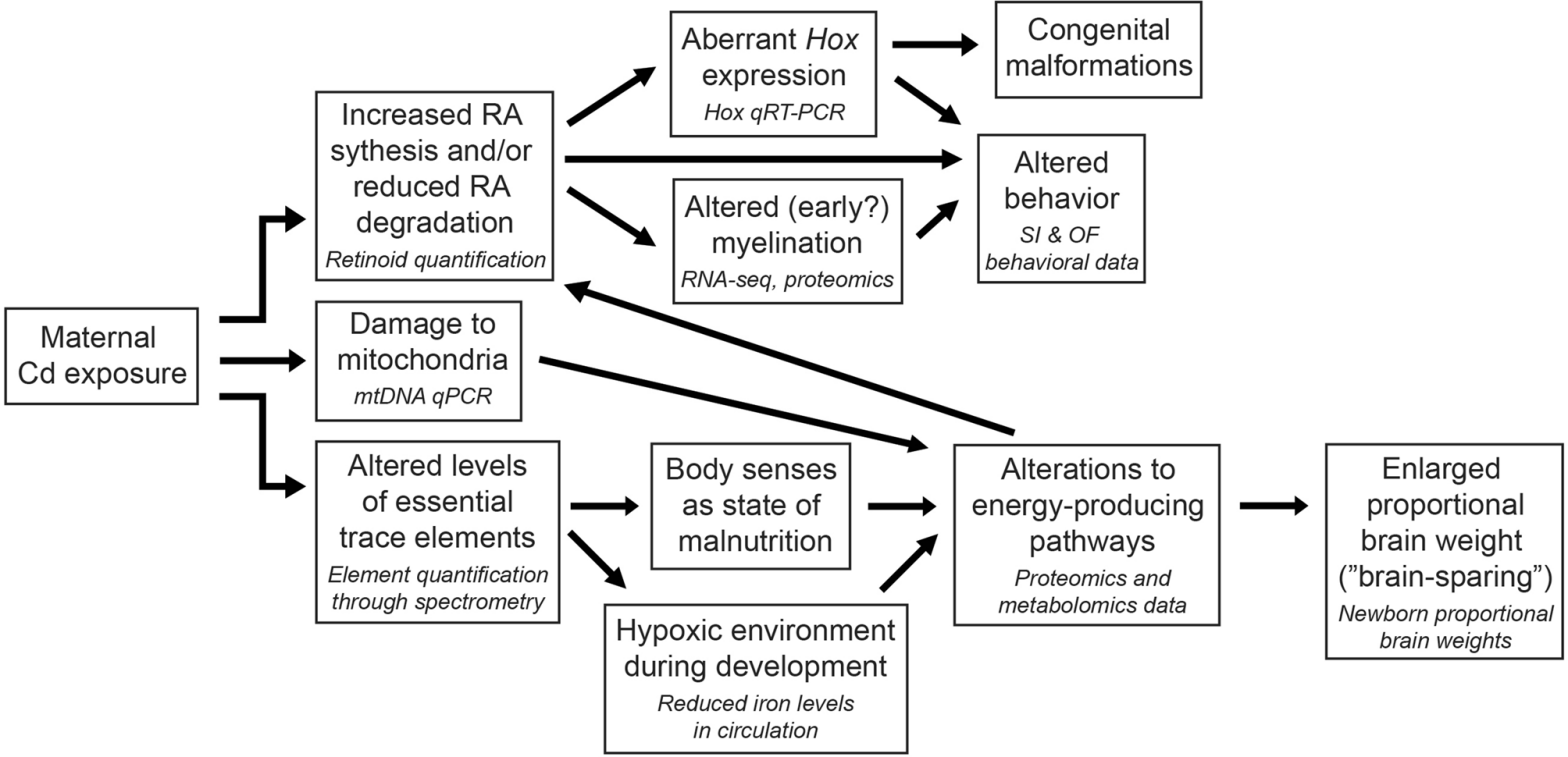
Proposed mechanisms and hypotheses linking maternal Cd exposure to altered neurodevelopment. We propose that maternal Cd exposure increases retinoic acid (RA) synthesis and/or degradation in the fetal brain. This leads to aberrant *Hox* expression, altered (early onset of) myelination, and altered behavior. Aberrant *Hox* expression may explain the previously known role of Cd as a teratogen. Additionally, we propose that maternal Cd exposure damages the mitochondria in the fetal brain and therefore impacts energy production in the brain. Metabolites produced during this process may have feedback on RA production. Finally, we propose that maternal Cd exposure alters levels of essential trace elements, which is sensed by the body as a state of malnutrition and may lead to a hypoxic environment during development due to the significantly reduced levels of iron in circulation. This also alters energy production in the brain and may explain the enlarged proportional brain weights at birth, consistent with the phenomenon known as “brain-sparing”. These hypotheses lay the foundation for future mechanistic work to elucidate molecular pathways occurring at each arrow. Data described here or in Hudson et al.^15^ that support the hypotheses are italicized in smaller text below the corresponding hypothesis in each box.

Exposure to Cd during early life is associated with impaired neurodevelopment and behavior^18,19,21–23^, but the underlying molecular mechanisms responsible for these changes are poorly understood. In the present study, we utilized a mouse model of maternal Cd exposure and demonstrated that maternal exposure alone is sufficient to program altered behavior among F_1_ mice during adulthood. Behavioral endpoints for both males and females showed considerable variability within each group, which in females may partly be explained by not controlling for estrus cycle stage. Nonetheless, maternally-exposed mice overall demonstrated perturbation of anxiety-related behavior. To inform on the molecular mechanisms underlying these behavioral defects, we identified exposure-associated transcriptomic, proteomic, and metabolomic changes in the brain at birth. Together, these data suggest that maternal Cd exposure may cause premature myelination in the brain and inappropriate regulation of *Hox* gene expression. These data also suggest that Cd perturbs RA signaling in the developing brain, which we subsequently confirmed with targeted analyses. A summary of perturbed pathways identified in this work and our subsequent hypotheses of mechanisms that may link maternal Cd exposure to altered neurodevelopment and behavior are shown in Fig. 8. This work provides a foundation for further studies to understand the mechanisms of maternal Cd exposure. Our mouse model also provides the opportunity to study the adverse effects of Cd on other organ systems. Here we report only impacts on neurodevelopment and behavior, but have previously shown that maternal Cd exposure can affect cardiovascular development and program hypertension in a subset of adult F_1_ animals^15^.

Two doses of Cd were used in this study: 1 ppm Cd, which reflects a realistic level of routine Cd exposure considering the average levels of Cd naturally occurring in the Earth’s crust, and 50 ppm Cd, as this dose results in circulating blood Cd levels consistent with those found in people living in heavily Cd polluted areas^15^. In some of our experiments, the low dose of Cd had more pronounced effects than the high dose; this supports the emerging frequency of non-monotonic dose-responses in toxicological studies^48^. Rather than starting exposure at conception, our mouse model began maternal Cd exposure prior to conception in order to more closely reflect typical patterns of chronic human exposure. Additionally, our mouse model used ingestion of Cd-laced water, rather than injection, to more closely reflect a common route of Cd exposure in humans.

In rodent brains, myelination is an event that primarily occurs during postnatal development^29^. Our RNA-seq and proteomic analyses are suggestive of an earlier onset of myelination due to maternal Cd exposure. Twelve of the fifteen maternal Cd-associated DEGs are involved in myelination or oligodendrocyte differentiation during myelination, and two classical myelin markers (*Mbp* and *Mag*) were significantly increased in newborn pup brains in a dose-dependent manner. In addition, 20 proteins that are part of the myelin proteome were found to be significantly increased in abundance due to 50 ppm maternal Cd exposure. Finally, we previously showed that maternal Cd exposure increases circulating fetal copper levels^15^, an element that is required for myelination^49^. To date, the link between Cd and myelin has only been made following postnatal or adult exposure to Cd and suggests a negative effect on myelination^50,51^. However, one study in rats that induced a delay in early postnatal myelination through lysophosphatidylcholine injection into the ventral hippocampus of 10 day old rat pups noted opposite behavioral effects to those seen in this study (i.e. decreased activity and increased anxiety-related behaviors)^52^. There is emerging evidence that myelin plasticity, remodeling, and altered developmental myelination affects behavior^35,53,54^, thereby supporting a potential mechanistic link through which maternal Cd exposure impacts offspring behavior. Myelin proteins and other molecular markers of myelination can be found in the rodent brain as early as 7 days postnatally^29^, but myelin is difficult to detect using histological approaches, consistent with our inability to visualize myelin in newborn maternally-exposed pup brains with black-gold staining (data not shown).

We also show that *Hox* gene expression is inappropriately regulated following maternal Cd exposure. The *Hox* gene family is conserved across metazoan species and is essential during development to confer anterior to posterior identity to tissues. *Hox* gene expression patterns are uniquely linked to their position along their respective chromosomes: 3’ *Hox* genes are expressed in the anterior end of the embryo, while 5’ *Hox* genes are expressed more posteriorly. *Hox* expression is regulated in part by a RA gradient in the developing embryo. Mammals have four clusters of *Hox* genes (A, B, C, D), each cluster located on a different chromosome, and the genes are numbered 1–13 based on their 3’ to 5’ location within their cluster^44^. *Hox* 1–4 paralogs are expressed in the mammalian central nervous system, primarily in the developing hindbrain; the remaining *Hox* 5–13 paralogs are expressed in the spinal cord and other posterior tissues^55^. In this study, we have shown that expression of the *Hoxa* and *Hoxb* clusters is perturbed due to maternal Cd exposure, with regards to 2–4 paralogs that are normally expressed in the brain as well as more posteriorly expressed *Hox* genes. *Hox1-4* paralog expression in the developing central nervous system is essential for linking neurons involved with motor activity to their behavioral output^56^. *Hoxb8*, identified through RNA-seq as the most significantly differentially expressed gene in the brain due to maternal Cd exposure, has been shown to affect behavior; mutant mice display excessive and pathological grooming behaviors, and *Hoxb8* expression has been found in obsessive–compulsive disorder neural circuits in humans^55^. In vitro, direct exposure to Cd has been shown to induce expression of *Hoxb8*^57^. Additionally, altered *Hox* expression may explain how maternal Cd exposure can lead to congenital malformations^58,59^, as mutations in *Hox* genes can lead to drastic developmental malformations^60^.

The brain is the most energy expensive organ in the body. Despite comprising only 2% of the body weight, the brain utilizes 20% of the body’s energy while at rest. Proper circulating levels of nutrients such as glucose and oxygen are essential to meet this demand^61^. We have previously shown that maternal Cd exposure leads to systemic alterations of essential trace elements in the offspring at birth, including a severe iron deficiency, which suggests that cellular energy pathways may be negatively impacted as a result^15^. The proteomic analysis and reduced mitochondrial content seen in newborn brains here are consistent with perturbed cellular energy and hypoxic conditions due to maternal Cd exposure. Because of the important role of the brain, this organ is typically spared during intrauterine growth restriction. In response to hypoxic or nutrient-poor conditions, the fetus adapts by rerouting its circulation to conserve oxygen and nutrient supply for the brain^62^. This brain-sparing phenomenon may at least partially explain why maternal Cd exposure did not significantly alter the raw weight of brains at birth, despite the mice showing a reduction in birth weight^15^.

Using a discovery-based metabolomics approach, we identified three significantly increased metabolites in newborn brains due to 50 ppm maternal Cd exposure. Two of these metabolites, 2-hydroxyvaleric acid and 2-hydroxycaproic acid, are both medium chain alpha-hydroxy fatty acids that are not well studied in the context of mammalian neurodevelopment. Elevated levels of 2-hydroxyvaleric acid are associated with lactic acidosis^63^, a condition characterized by excess lactic acid^64^ and consistent with our hypothesis of elevated lactate in the brain due to a hypoxic environment caused by maternal Cd exposure. 2-hydroxyvaleric acid is also used in a staining method for measuring tissue-specific lactate dehydrogenase activity^65^, and the elevation in 2-hydroxyvaleric acid would be consistent with the elevated Ldha identified through our proteomic analysis and further supporting our hypothesis regarding elevated lactate. Elevated 2-hydroxycaproic acid levels are seen in response to iron depravation^66^ and as a biomarker for cardiovascular mortality^67^, consistent with our previously published work^15^. 2-hydroxycaproic acid is an inhibitor of aminoacylase 1^67^ (ACY1); ACY1 is strongly expressed in the brain and its deficiency, though rare, is characterized by impaired neurodevelopment^68^. The third metabolite identified by metabolomics (xanthine) has been studied in greater detail. Xanthine is a purine derivative involved in a number of enzymatic reactions and is potentially toxic^63^. Xanthine accumulates in mitochondrial dysfunction^69^ and is produced in the Mitochondrial DNA Depletion Syndrome disease pathway, which is characterized by decreased levels of mtDNA^70^ as was seen in this study as a result of maternal Cd exposure. Molybdenum cofactor deficiency is a severe neurological disorder that results from buildup of xanthine due to a nonfunctional molybdenum-dependent cofactor that is required for certain enzymes^63,71^; we previously showed that maternal Cd exposure leads to a significant decrease in molybdenum in newborns^15^, and molybdenum cofactor production may further be impaired by a maternal Cd-induced iron deficiency^15,72^. The maternal Cd-induced increase in xanthine and the increase in hypoxanthine–guanine phosphoribosyltransferase (Hprt, as identified through our proteomic analysis) may be suggestive of an overall impairment or alteration to purine metabolism, which is emerging as an important metabolic pathway for proper brain function and development^73^. Finally, two enzymes involved in xanthine metabolism (xanthine oxidase and xanthine dehydrogenase) may play a role in RA metabolism and synthesis^74,75^.

Using multiple -omic approaches, we identified RA signaling as a candidate pathway that is perturbed by maternal Cd exposure in the developing brain. To test this, we quantified retinoid species and confirmed increased levels in newborn brains as a result of maternal Cd exposure. It is possible that xanthine was increased in maternally-exposed brains due to xanthine oxidase and/or xanthine dehydrogenase being sequestered for RA synthesis, though this will need to verified experimentally. RA is required for the onset of myelination^31^ and *Hox* gene expression is controlled in part by a rostrocaudally-decreasing gradient of RA along the body axis^56^. Abnormal levels of RA during development, either excess or reduced, lead to embryological defects, affect hindbrain development, and alter *Hox* gene expression^55^. Excess RA during development can lead to profound behavioral abnormalities and mental retardation in the offspring^47^. In vitro and adult exposure to Cd has been shown to induce RA signaling, and Cd teratogenicity is suspected to be at least in part due to increased RA as a result of disruption to RA-metabolizing genes^76^, consistent with the data in this study.

The transcriptomic, proteomic, and metabolomic analyses of newborn brains are likely to be affected by the cellular heterogeneity of whole brains. Brain heterogeneity can bias sequencing analyses^77^ and may exclude region- or cell-specific changes and limit differential expression analysis to abundant transcripts, consistent with the low number of differentially expressed genes identified in this study. Methods such as surrogate variable analysis (SVA) and remove unwanted variation (RUV) can potentially control for sources of heterogeneity ^78,79^. However, one of our key findings is that genes involved in myelination are altered in abundance and given that this is a biological process that occurs throughout the brain, we report the global results in this study without implementing a statistical method to control for heterogeneity. The proteomic analysis can only quantify levels of proteins that are abundant in tissue, and therefore can miss detection of proteins found at lower levels in the cell (e.g., transcription factors^80^). This may explain the lack of overlap between genes identified as differentially expressed through RNA-seq and the proteins found to be differentially abundant. Despite these caveats, we were able to detect an enrichment for RA-associated terms in both the transcriptomic and proteomic datasets, which was supported by the retinoid species quantification. In addition, 5 proteins with roles in cellular energy metabolism that were found to be significantly increased in abundance here (Ldha, Pfkl, Aldoc, Anxa2, Aldoa) were also found to be significantly increased at the level of gene expression in newborn hearts as a result of maternal Cd exposure^15^, suggesting that maternal Cd exposure-associated alterations to cellular energy metabolism may be apparent in multiple organs.

In summary, we have shown global changes to the transcriptome, proteome, and metabolome in newborn mouse brains as a result of maternal Cd exposure and have generated multiple hypotheses for future mechanistic work to link maternal Cd exposure to altered neurodevelopment (Fig. 8). As a proof of principle that our multiple -omics approach can identify novel candidate pathways perturbed by maternal Cd exposure, we demonstrated maternal Cd-associated changes to retinoid species in the brain, providing empirical support for the alterations to RA signaling suggested by transcriptomic and proteomic data. Overall, our results support findings of other studies that suggest a neurotoxic effect of early life Cd exposure and provide new insights into understanding the link between maternal Cd exposure and impaired neurodevelopment.

## Materials and methods

### Animal husbandry, Cd exposure, and tissue collection

Animal work and tissue collection were performed as described previously^15^. Briefly, 5- to 7-week-old C57BL/6J (‘B’) and CAST/EiJ (‘C’) females (the F_0_ generation) were exposed through their drinking water to 0 ppm, 1 ppm, or 50 ppm Cd in the form of CdCl_2_ (Sigma-Aldrich, catalog number 202908) for a period of 5 weeks, then mated with a male of the opposite strain. Cd exposure continued throughout pregnancy and was discontinued at litter birth. A hybrid mating scheme (the F_1_ generation) was employed to enable analyses of allele-specific gene expression and DNA methylation for a separate study (Hudson & Cowley, manuscript in preparation). The genotypes of F_1_ mice are referred to as ‘B × C’ (B mother × C father) and ‘C × B’ (C mother × B father). F_1_ animals were sacrificed and dissected within 24 h of birth or at 6 months of age; sample sizes can be found in a previous publication that studied the effect of maternal Cd exposure on cardiovascular features in these mice^15^. F1 mice dissected within 24 h of birth are referred to as ‘newborn’. A detailed account of housing conditions and sample sizes used in this study can be found in a previous publication^15^.

All animal work was approved by the North Carolina State University (NCSU) Institutional Animal Care and Use Committee, under protocol 16–045-B. All experiments were conducted in accordance with the Guiding Principles in the Use of Animals in Toxicology.

### Behavioral assessments

F_1_ mice were subjected to two behavioral tests at approximately 5.5 months (+ /- 2 weeks) of age over two consecutive days: open field (OF) and social interaction (SI). Behavioral testing was performed as previously described^81,82^. Females were not controlled for stage of estrous cycle. Boxes were disinfected between the testing of each subject. All testing was performed during the afternoon between 3 pm-6 pm under dimmed lighting in a secluded procedure room, video recorded for 30 min, and scored using TopScan software (Clever Sys Inc.). A randomly selected subset of 2 videos per treatment group and per sex was scored by hand to validate TopScan scoring.

SI was performed in the same box one day after OF testing using an unexposed B mouse of the same sex and of similar age. Pure B strain mice that were the same sex as the hybrid F_1_ test animal were used as novel test mice in place of B × C or C × B hybrids for the SI experiments due to the increased aggression shown by hybrid male mice to each other.

Sample sizes used in behavioral experiments, source data, and statistical summaries for all behavioral parameters measured can be found in Supplementary Table 1. The number of litters represented in animals subjected to behavioral testing can be found in Supplementary Table 1 of a previous publication^15^, as the animals described here were subjected to blood pressure testing 1–2 weeks after behavioral testing. A one-way analysis of variance (ANOVA) followed by a post-hoc Tukey test using R software was performed on the data from the OF and SI experiments for the four control groups (B × C females, B × C males, C × B females, C × B males) to identify any significant differences in behaviors of unexposed mice. All other statistical analyses were performed using an ANOVA followed by a post-hoc Dunnett’s multiple comparison test using R software, comparing animals of the same sex and genetic background from the 1 ppm and 50 ppm groups to 0 ppm controls.

### Histology

Newborn whole brains or heads were immediately immersed in 10% neutral buffered formalin (NBF), fixed for 48 h, transferred to a 30% sucrose solution until sunk, then submitted to the NC State’s College of Veterinary Medicine Histology Laboratory. There, the brains were either embedded in paraffin and stained with Hematoxylin & Eosin (H&E), or the heads were frozen and cryosectioned for Black Gold II staining.

Formalin fixed paraffin embedded (FFPE) brains were processed routinely, oriented for 5 µm sectioning in the coronal plane, and stained with H&E stain. H&E staining was performed on every fifth serial coronal section taken from the rostral to caudal end to reduce the number of slides processed. A veterinary board-certified pathologist (ACVP) identified anatomical landmarks throughout the brain on the slides in order for accurate comparisons between the same regions across individuals.

Frozen heads were cryosectioned on the coronal plane at a thickness of 20 µm to obtain sections representing the prefrontal cortex. Further processing and black-gold staining was performed on slides containing comparable regions in the prefrontal cortex across individuals according to manufacturer directions (Millipore Black-Gold II Myelin Staining Kit, catalog number AG105).

### Nucleic acid isolation

Frozen whole brains obtained from eight 0 ppm, eight 1 ppm, and eight 50 ppm newborn female B × C pups representing four litters per exposure group were pulverized using a hammer and liquid nitrogen. 7–10 mg of tissue was then used to extract DNA and RNA simultaneously using the AllPrep DNA/RNA/miRNA kit (Qiagen, 80204). RNA was extracted using the recommended protocol for fatty tissues. RNA and DNA were quantified using a Nanodrop 2000. RNA purity and size integrity were determined at the NCSU Genomic Sciences Laboratory (GSL) using an Agilent 2100 Bioanalyzer with an RNA 6000 Nano Chip (Agilent Technologies).

### RNA-seq

Total RNA from the brains of four 0 ppm and four 50 ppm maternally exposed female F_1_ mice (each individual representing a different litter) were submitted to the NCSU GSL for indexed library construction and sequencing as described previously^15^.

The quality of raw RNA-seq data was assessed using the FastQC application and the initial 10 bases with poor quality were trimmed. Alignment was performed using STAR short read aligner^83^ to the respective mouse strain (C57BL/6J, CAST/EiJ) reference genomes. C57BL/6J and CAST/EiJ RNA-seq data were aligned to mm38 version 87 and CAST/EiJ version 1.86 reference genome downloaded from the Ensembl website, respectively. The number of reads mapped to a genome feature was determined using htseq-count script from HTSeq python package^84^. Independent analyses were conducted for the two strains. The two raw count data matrices were imported to R statistical computing environment for further analysis^85^. Genes that had no count in more than 2/3 of the replicate samples were excluded from the analysis. Data normalization based on dispersion and differential analysis was conducted using the DESeq2 R package^86^. A generalized linear model (~ Treatment) was fitted between the expression count and a treatment group (0 ppm or 50 ppm maternal Cd exposure). Finally, a list of differentially expressed genes was generated between 50 ppm vs. 0 ppm maternal Cd exposure after applying Benjamini–Hochberg multiple testing correction^87^ (p adj < 0.05). The entire dataset of all genes with detectable and good quality reads aligned to both genomes can be found in Supplementary Table 2. The heatmap of the 15 significantly differentially expressed genes shown in Fig. 3 was generated through http://www.heatmapper.ca/ using average linkage clustering method and Pearson distance measurement method.

Data generated by RNA-seq in this study are deposited in Gene Expression Omnibus (GEO), accession number GSE174433.

### qRT-PCR and qPCR

100 ng of total RNA extracted as described above from 24 B × C female newborn F_1_ brains was used to synthesize first strand cDNA as described previously^15^; the 8 samples used in RNA-seq were included in these 24 samples. qRT-PCR was performed on a Real-Time 7300 machine (Applied Biosystems) using SsoAdvanced Universal SYBR Green Supermix (Bio-Rad, 1725271) using the conditions and methods of analysis described previously^15^. The primer sequences used for the *Hox* genes in Fig. 4 are provided in Supplementary Table 6. Additional *Hox* genes were tested, but did not have consistent detectable amplification in at least one of the three maternal Cd dose groups. We defined consistent detectable amplification as at least 3 of the 8 pups’ cDNA samples per treatment group generating C_t_ values for each technical replicate (i.e. for one pup, generating three C_t_ values for the three technical replicates, and this occurring for at least 3 out of 8 pups in a group).

The ΔΔC_t_ method of qRT-PCR gene expression analysis requires a comparison between a sample’s ΔC_t_ value to the average ΔC_t_ value of the control group, where ΔC_t_ = C_t_(gene of interest) − C_t_(reference gene). Due to undetectable amplification of several *Hox* genes in multiple control samples (which resulted in undetermined C_t_ values in up to 6 of the 8 control samples), the average ΔC_t_ value of the 1 ppm group was used as the “control” ΔC_t_ value for calculating the ΔΔC_t_ value. The expression values are therefore given relative to the 1 ppm group (Fig. 4). Samples with undetermined C_t_ values were then given a relative expression value of 0%.

DNA was extracted from 24 B × C female newborn F_1_ brains through the Qiagen kit described above. Mitochondrial DNA (mtDNA) was quantified by qPCR as previously described^88,89^.

To provide greater transparency and allow for better reproducibility of results, this study was performed in compliance with MIQE standards^90^ (see Supplementary Table 7).

### Proteomics

Frozen homogenized whole brains obtained from newborn B × C maternally exposed female pups were submitted to the Molecular Education, Technology, and Research Innovation Center (METRIC) at NCSU for proteomic analysis. The brain samples used in the proteomic analysis originated from the four 0 ppm and four 50 ppm females represented in the RNA-seq analysis; additionally, four 1 ppm females representing four different litters were used for proteomic analysis.

Samples were quantitated for protein content using a Pierce bicinchoninic acid (BCA) kit and normalized to 50 μg of protein by diluting the appropriate amount of sample in 50 mM ammonium bicarbonate with 1% (w/w) sodium deoxycholate. Normalized samples were reduced with dithiothreitol (DTT) and alkylated with iodoacetamide (IAM) to break disulfide bonds and prevent reformation. Following this, a filter aided sample preparation (FASP) protocol^91,92^ was used to purify the samples which were then treated with trypsin at a 50:1 protein:trypsin concentration. Samples were incubated for 4 h and then aliquoted for LC–MS analysis.

Chromatographic separation was achieved using a Thermo Scientific EASY nLC 1200 system (Bremen, Germany). Pico-frit columns were purchased from New Objective (Woburn, MA) and bomb packed to a length of 20–30 cm with reverse phase ReproSil-Pur 120 C-18- AQ 3 µm particles (Dr. Maisch, Germany). Two microliters of sample was injected and subsequently separated using a gradient of mobile phase A (98% water, 2% acetonitrile, and 0.1% formic acid) and mobile phase B (80% acetonitrile, 20% water 0.1% formic acid). The LC method consisted of a 120 min gradient with a linear ramp from 0% B to 40% B, a 1 min ramp to 100% B which was held for 6 min (123–129 min), followed by equilibration of the column at 0% B (130–140). A flow rate of 300 nL/min was used.

Orbitrap tandem mass spectrometry was performed using a Thermo Scientific Q-Exactive HF (Bremen, Germany) in a top 20 data dependent acquisition (DDA) mode, where the 20 most abundant precursors are selected for fragmentation per full scan. MS1 and MS2 scans were performed at a resolving power of 120,000 and 15,000 at m/z 200, respectively. A dynamic exclusion window of 20 s was used to avoid repeated interrogation of abundant species. Automatic gain control (AGC) was 1e6 and 1e5 for MS1 and MS2 scans, respectively. Samples were run in random order, and a quality control BSA digest was run every fifth injection to ensure proper LC–MS/MS reproducibility.

Resulting raw data were loaded into Proteome Discoverer (version 2.0). Spectrum files were run through the spectrum selector node to appropriately identify peaks and this data was collated using the Sequest HT node and aligned with a FASTA file which contained all proteins indexed by Uniprot and assigned a taxonomy ID of 10,090 (mus musculus). Oxidation (dynamic) and carbamidomethylation (static) modifications were considered in Sequest HT with a maximum of 2 potential missed cleavage sites and peptide lengths between 6 and 144 amino acids. The Percolator node was used for false discovery rate (FDR) calculations.

Data was then filtered to remove the following: abundance ratio adj. p-value (50 ppm vs 0 ppm) > 0.05, # unique peptides > 1, Abundances (grouped) CV% 50 ppm > 20, Abundances (grouped) CV% 0 ppm > 25, proteins with a combination of low PEP score and CV% > 15, PEP score < 7, and proteins that had a ‘Found in Sample’ value of any value lower than ‘High’ for at least 2 individuals.

Proteomics raw data can be found in Supplementary Table 3.

### RNA-seq and proteomic enrichment analyses

The lists of 15 significantly differentially expressed genes and 46 proteins significantly different in abundance in the 50 ppm group compared to controls were submitted for separate Enrichr analyses^93,94^ at http://amp.pharm.mssm.edu/Enrichr/ . Databases with relevant terms were considered and the comprehensive lists can be found in Supplementary Tables 2 and 4.

### Lactate quantification and metabolomics

Four homogenized newborn B × C female brains were tested for each of the three treatment groups, and each sample was tested twice. One 50 ppm sample was destroyed during processing, resulting in a final n = 3 for that group. To each mouse brain sample, 100 µL of acidified mobile phase containing ITSD (50 ug/mL 13C3-sodium lactate, Cambridge Isotope Laboratories) and 500 µL of cold methanol were added prior to homogenization using an Omni tissue homogenizer with disposable tips. After 10 s of homogenization, the samples were allowed to sit at −20 °C for 30 min before centrifugation for 15 min (4 °C, 3000×*g*) for protein precipitation. The supernatant was removed and transferred to a fresh 1.5 mL Eppendorf tube and taken to dryness on a SpeedVac. The samples were then resuspended in 100 µL mobile phase A and centrifuged for 15 min (4 °C, 3000×*g*). The supernatant was transferred directly to an LCMS autosampler vial for analysis. Quality control (QC) samples were prepared by pooling and mixing equal volumes of each sample. The QC sample and blank samples were injected at regular intervals throughout the sequence. For quantification of lactate, a 100 ug/mL stock solution of sodium lactate (Cambridge Isotope Laboratories) was prepared using acidified mobile phase containing ITSD (50 ug/mL 13C3-sodium lactate, Cambridge Isotope Laboratories). Seven calibration standards ranging from 3.2 ng/mL to 50 µg/mL were prepared by serially diluting the working standard solution with acidified mobile phase containing ITSD. Chromatographic separation was achieved using a Waters Acquity BEH C18 column (2.1 × 100 mm) holding for 5 min at 100% mobile phase A (5% MeOH in water + 0.15% formic acid) followed by gradient elution (mobile phase B 100% MeCN) to 95% mobile phase B (flow rate 350 µL/min, 2 µL injections in duplicate). High resolution mass spectrometry data was acquired using a Thermo Orbitrap ID-X mass spectrometer in negative mode (spray voltage 3.0 kV, vaporizer temperature 300 °C) with a mass range of m/z 70–800 and a dwell time of 0.6 s based on the chromatographic peak width of 6 s allowing 10 scans across each peak for accuracy in quantification. MS^1^ data was collected with a resolution of 120,000 and an AGC target of 2.0e5. MS^2^ data was collected for global metabolite profiling (AGC target 1.0e5, resolution 30 K, and stepped HCD collision energy of 20, 35, 50).

#### Targeted data processing

Peak integration and quantification was performed in TraceFinder 4.1. A standard curve for lactate was constructed using extracted ion chromatogram peak areas from MS1 data and the slope of the curve was calculated in reference to the ISTD using the area of the peak divided by the internal standard peak area with a 1/x weighting. The concentration of lactate in the study samples was calculated in an identical manner relative to the regression line. The lactate calibration curve had an R^2^ value of 0.9994 for the linear range of 80 ng/mL to 50 ug/mL.

#### Untargeted data processing

##### *Preprocessing and annotation in CD 3.0*

Raw data files were uploaded into Compound Discoverer 3.0 (Thermo Fisher Scientific) and processed using a workflow to find and identify differences between samples. This workflow performed retention time alignment, unknown compound detection and compound grouping across all samples. Elemental compositions were predicted for all compounds and chemical background was hidden using blank samples. Compound annotations were assigned using ddMS2 data in comparison with the mzCloud database (Thermo Fisher Scientific) as well as molecular formula comparisons with selected ChemSpider databases (KEGG, HMDB, Mass Bank) which were further ranked using the mzLogic algorithm (Thermo Fisher Scientific).

##### *Compound annotations*

A total of 1,471 compounds were identified in the data set. Compounds were filtered in order to exclude background resulting in 1,217 compounds of which 162 were annotated in Compound Discoverer. Manual curation of these 162 compounds to remove duplicate and erroneous annotations resulted in 101 compounds.

##### *Statistical analysis in MetaboAnalyst*

The filtered data set (99 compounds) with peak areas per file were formatted for further statistical analysis in MetaboAnalyst^95^. Sample normalization, data transformation, and data scaling were set to normalization by sum, log transformation, and pareto scaling, respectively. Results were analyzed using univariate (T-test/ANOVA, Fold Change, Volcano Plot, Correlation Analysis) and multivariate (PCA, PLS-DA) statistics as well as by hierarchical clustering (Heatmap) and supervised feature selection (Random Forest). Normalized data for each sample was exported from MetaboAnalyst into an excel file and the mean value of duplicate values for each sample for the 99 compounds was calculated. Outliers, ANOVAs, and post-hoc Dunnett’s test were calculated as described below in the Statistical Analysis section, with a final step of Benjamini–Hochberg correction for multiple testing of 99 metabolites. Metabolomics data can be found in Supplementary Table 5.

### Retinoid quantification

Whole brains of newborn B × C maternally-exposed pups were removed within 30 s of pup sacrifice under yellow light, placed in a black 1.7 mL microcentrifuge tube to prevent exposure to white light, then immediately weighed and flash frozen on dry ice. Sample sizes were as follows: sixteen 0 ppm females, five 1 ppm females, eleven 50 ppm females, eleven 0 ppm males, seven 1 ppm males, and eight 50 ppm males.

Retinoid concentrations were measured as described previously^96–98^. Briefly, an average of 85.2 ± 9.2 mg (mean ± s.d.) (range 67.1—117.1 mg) per sample of brain was homogenized in ground glass homogenizers in 1.0 mL normal saline (0.9% NaCl). Extraction of retinoids was performed under yellow lights using a two-step liquid–liquid extraction that has been described in detail previously using 4,4-dimethyl-RA as an internal standard for RA and retinyl acetate as an internal standard for retinol and total retinyl ester^96,98^. RA levels were determined by liquid chromatography-multistage tandem mass spectrometry (LC-MRM3), which is an LC–MS/MS method utilizing two distinct fragmentation events for enhanced selectivity^96^. RA was quantified using a Shimadzu Prominence UFLC XR liquid chromatography system (Shimadzu, Columbia, MD) coupled to an AB Sciex 6500 + QTRAP hybrid triple quadrupole mass spectrometer (AB Sciex, Framingham, MA) using atmospheric pressure chemical ionization (APCI) operated in positive ion mode as previously described^96^. Retinol and retinyl ester were quantified via HPLC–UV using an AQUITY H-Class UPLC with a PDA detector (Waters Corporation, Milford, MA) operated in single wavelength monitoring mode at 325 nm according to previously published methodology^97,98^. Retinoids in tissues are expressed as mol/g tissue.

### Statistical analysis

Unless otherwise noted, all statistical analyses were performed using an ANOVA followed by a post-hoc Dunnett’s multiple comparison test using R software (version 4.0.2, https://cran.r-project.org), comparing animals of the same sex and genetic background from the 1 ppm and 50 ppm groups to 0 ppm controls. Outliers, as defined by a point which falls more than 1.5 times the interquartile range above the third quartile or below the first quartile, were omitted from the analysis if found to be present. Data are presented as the mean ± standard error of the mean. *p < 0.05, **p < 0.01, ***p < 0.001. Source data from each figure and data referenced in the main text can be found in Supplementary Table 8.

## Supplementary Information

Supplementary Information 1.

Supplementary Information 2.

Supplementary Information 3.

Supplementary Information 4.

Supplementary Information 5.

Supplementary Information 6.

Supplementary Information 7.

Supplementary Information 8.

Supplementary Information 9.

Supplementary Information 10.

Supplementary Information 11.

## References

[1] Caserta, D., Graziano, A., Monte, G.L.O., Bordi, G. & Moscarini, M. Heavy metals and placental fetal-maternal barrier: A mini-review on the major concerns. *Eur. Rev. Med. Pharmacol. Sci.* **17**, 2198–2206 (2013). 23893187

[2] Gundacker C, Hengstschlager M (2012). The role of the placenta in fetal exposure to heavy metals. Wien. Med. Wochenschr..

[3] W.H.O. Ten chemicals of major public health concern. https://www.who.int/ipcs/assessment/public_health/chemicals_phc/en/ (2018).

[4] WHO. EXPOSURE TO CADMIUM : A MAJOR PUBLIC HEALTH CONCERN. (2010).

[5] Järup L, Åkesson A (2009). Current status of cadmium as an environmental health problem. Toxicol. Appl. Pharmacol..

[6] Ferramola ML, Antón RI, Anzulovich AC, Giménez MS (2011). Myocardial oxidative stress following sub-chronic and chronic oral cadmium exposure in rats. Environ. Toxicol. Pharmacol..

[7] Hu, H. *et al.* RNA-seq identifies key reproductive gene expression alterations in response to cadmium exposure. *Biomed. Res. Int.* **2014** (2014). 10.1155/2014/529271 PMC4058285 24982889

[8] Filipic, M. Mechanisms of cadmium induced genomic instability. *Mutat. Res. Fundam. Mol. Mech. Mutagen.* **733**, 69–77 (2012). 10.1016/j.mrfmmm.2011.09.002 21945723

[9] Hyder O (2013). Cadmium exposure and liver disease among US adults. J. Gastrointest. Surg..

[10] Satarug S, Garrett SH, Sens MA, Sens DA (2010). Cadmium. Environmental Exposure, and Health Outcomes..

[11] WHO. Global status report on noncommunicable diseases 2010. (2011).

[12] Tellez-Plaza M (2012). Cadmium exposure and all cause and cardiovascular mortality in the US general population. Environ. Health Perspect..

[13] Chandravanshi Lalit Shiv Kunal, K. S. Developmental toxicity of cadmium in infants and children: A review. *Environ. Anal. Health Toxicol.* **36**, e20210030 (2021). 10.5620/eaht.2021003 PMC8207007 33730790

[14] Tchounwou, P. B., Yedjou, C. G., Patlolla, A. K. & Sutton, D. J. Heavy Metals Toxicity and the Environment. 1–30 (2014). 10.1007/978-3-7643-8340-4. 10.1007/978-3-7643-8340-4_6 PMC4144270 22945569

[15] Hudson KM, Belcher SM, Cowley M (2019). Maternal cadmium exposure in the mouse leads to increased heart weight at birth and programs susceptibility to hypertension in adulthood. Sci. Rep..

[16] Ronco AM, Urrutia M, Montenegro M, Llanos MN (2009). Cadmium exposure during pregnancy reduces birth weight and increases maternal and foetal glucocorticoids. Toxicol. Lett..

[17] del Díaz CM (2014). Effect of a single dose of cadmium on pregnant Wistar rats and their offspring. Reprod. Domest Anim..

[18] Wang Y (2016). Effects of prenatal exposure to cadmium on neurodevelopment of infants in Shandong, China. Environ. Pollut..

[19] Tian, L. L. *et al.* Effects of gestational cadmium exposure on pregnancy outcome and development in the offspring at age 4.5 years. *Biol. Trace Elem. Res.* **132**, 51–59 (2009). 10.1007/s12011-009-8391-0 19404590

[20] Liu, J. *et al.* Interaction of prenatal bisphenols, maternal nutrients, and toxic metal exposures on neurodevelopment of 2-year-olds in the APrON cohort. *Environ. Int.* **155**, 106601 (2021). 10.1016/j.envint.2021.106601 33962233

[21] Grawé KP, Teiling-Gårdlund A, Jalkesten E, Oskarsson A (2004). Increased spontaneous motor activity in offspring after maternal cadmium exposure during lactation. Environ. Toxicol. Pharmacol..

[22] Minetti A, Reale CA (2006). Sensorimotor developmental delays and lower anxiety in rats prenatally exposed to cadmium. J. Appl. Toxicol..

[23] Tian, J. *et al.* Cadmium chloride-induced transgenerational neurotoxicity in zebrafish development. *Environ. Toxicol. Pharmacol.* **81**, 103545 (2021). 10.1016/j.etap.2020.103545 33171223

[24] Zhang YM, Liu XZ, Lu H, Mei L, Liu ZP (2009). Lipid peroxidation and ultrastructural modifications in brain after perinatal exposure to lead and/or cadmium in rat pups. Biomed. Environ. Sci..

[25] Ishitobi H, Mori K, Yoshida K, Watanabe C (2007). Effects of perinatal exposure to low-dose cadmium on thyroid hormone-related and sex hormone receptor gene expressions in brain of offspring. Neurotoxicology.

[26] Mori K (2006). Effects of perinatal exposure to low doses of cadmium or methylmercury on thyroid hormone metabolism in metallothionein-deficient mouse neonates. Toxicology.

[27] Kaidanovich-Beilin, O., Lipina, T., Vukobradovic, I., Roder, J. & Woodgett, J. R. Assessment of Social Interaction Behaviors. *J. Vis. Exp.* **0**, 1–6 (2011). 10.3791/2473 PMC3197404 21403628

[28] Seibenhener, M. L. & Wooten, M. C. Use of the Open Field Maze to Measure Locomotor and Anxiety-like Behavior in Mice. *J. Vis. Exp.* **February**, 1–6 (2015). 10.3791/52434 PMC4354627 25742564

[29] Salvati S, Attorri L, Avellino C, Di Biase A, Sanchez M (2000). Diet, lipids and brain development. Dev. Neurosci..

[30] Thakurela S (2016). The transcriptome of mouse central nervous system myelin. Sci. Rep..

[31] Kazakova N (2006). A screen for mutations in zebrafish that affect myelin gene expression in Schwann cells and oligodendrocytes. Dev. Biol..

[32] Mengler L (2014). Brain maturation of the adolescent rat cortex and striatum: Changes in volume and myelination. Neuroimage.

[33] Fard, M. K. *et al.* BCAS1 expression defines a population of early myelinating oligodendrocytes in multiple sclerosis lesions. *Sci. Transl. Med.* **9**, (2017). 10.1126/scitranslmed.aam7816 PMC7116798 29212715

[34] Wicher G, Husic E, Nilsson G, Forsberg-Nilsson K (2013). Developmental expression of IL-33 in the mouse brain. Neurosci. Lett..

[35] Kaller MS, Lazari A, Blanco-Duque C, Sampaio-Baptista C, Johansen-Berg H (2017). Myelin plasticity and behaviour—connecting the dots. Curr. Opin. Neurobiol..

[36] Noronha AB (1989). Myelin-associated glycoprotein (MAG) and rat brain-specific 1B236 protein: Mapping of epitopes and demonstration of immunological identity. J. Mol. Neurosci..

[37] Zhang, S. *et al.* Sox2 is essential for oligodendroglial proliferation and differentiation during postnatal brain myelination and CNS remyelination. *J. Neurosci.* **38**, 1802–1820. 10.1523/JNEUROSCI.1291-17.2018 PMC5815459 29335358

[38] Monk KR, Talbot WS (2009). Genetic dissection of myelinated axons in zebrafish. Curr. Opin. Neurobiol..

[39] Petryniak MA, Potter GB, Rowitch DH, Rubenstein JLR (2007). Dlx1 and Dlx2 control neuronal versus oligodendroglial cell fate acquisition in the developing forebrain. Neuron.

[40] Boggs JM (2006). Myelin basic protein: a multifunctional protein. Cell. Mol. Life Sci..

[41] Brasko, C., Hawkins, V., De La Rocha, I. C. & Butt, A. M. Expression of Kir4.1 and Kir5.1 inwardly rectifying potassium channels in oligodendrocytes, the myelinating cells of the CNS. *Brain Struct. Funct.* **222**, 41–59 (2017). 10.1007/s00429-016-1199-8 PMC5225165 26879293

[42] Gravel, M., Trapp, B., Peterson, J. & Braun, P. E. CNP in Myelination - Overexpression alters oligodendrocyte morphogenesis. *Cell Biol. Pathol. Myelin* 75–82 (1997). 10.1007/978-1-4615-5949-8_8.

[43] Trimarco A (2014). Prostaglandin D2 synthase/GPR44: A signaling axis in PNS myelination. Nat. Neurosci..

[44] Deschamps J (2005). Developmental regulation of the Hox genes during axial morphogenesis in the mouse. Development.

[45] Jahn O, Tenzer S, Werner HB (2009). Myelin proteomics: Molecular anatomy of an insulating sheath. Mol. Neurobiol..

[46] Lee DC (2015). A lactate-induced response to hypoxia. Cell.

[47] Luo, T., Wagner, E., Crandall, J. E. & Dr̈ger, U. C. A retinoic-acid critical period in the early postnatal mouse brain. *Biol. Psychiatry* **56**, 971–980 (2004). 10.1016/j.biopsych.2004.09.020 15601608

[48] Lagarde F (2015). Non-monotonic dose-response relationships and endocrine disruptors: a qualitative method of assessment. Environ. Heal..

[49] Bhattacharya PT, Misra SR, Hussain M (2016). Nutritional aspects of essential trace elements in oral health and disease: An extensive review. Scientifica (Cairo)..

[50] Monaco A, Grimaldi MC, Ferrandino I (2016). Neuroglial alterations in the zebrafish brain exposed to cadmium chloride. J. Appl. Toxicol..

[51] Rai NK (2013). Exposure to As, Cd and Pb-mixture impairs myelin and axon development in rat brain, optic nerve and retina. Toxicol. Appl. Pharmacol..

[52] Makinodan M (2008). Lysophosphatidylcholine induces delayed myelination in the juvenile ventral hippocampus and behavioral alterations in adulthood. Neurochem. Int..

[53] Islam, R. & Kaffman, A. White-matter repair as a novel therapeutic target for early adversity. *Front. Neurosci.* **15**, 657693 (2021). 10.3389/fnins.2021.657693 PMC8062784 33897364

[54] Keogh CE (2021). Myelin as a regulator of development of the microbiota-gut-brain axis. Brain. Behav. Immun..

[55] Nolte, C. & Krumlauf, R. Expression of Hox genes in the nervous system of vertebrates. in *Madame Curie Bioscience Database [Internet]* 1–30 (Landes Bioscience, 2013).

[56] Dasen, J. S. & Jessell, T. M. *Chapter Six: Hox Networks and the Origins of Motor Neuron Diversity*. *Current Topics in Developmental Biology* vol. 88 (Elsevier Inc., 2009). 10.1016/S0070-2153(09)88006-X 19651305

[57] Nakagawa K, Lee MJ, Sasaki N, Hayashi C, Nishio H (2008). Cadmium exposure induces expression of the HOXB8 gene in COS-7 cells. Toxicol. Vitr..

[58] Holt D, Webb M (1987). Teratogenicity of ionic cadmium in the Wistar rat. Arch. Toxicol..

[59] Samarawickrama GP, Webb M (1979). Acute effects of cadmium on the pregnant rat and embryo-fetal development. Environ. Health Perspect..

[60] Quinonez SC, Innis JW (2014). Human HOX gene disorders. Mol. Genet. Metab..

[61] Andreone BJ, Lacoste B, Gu C (2015). Neuronal and vascular interactions. Annu. Rev. Neurosci..

[62] Cohen E, Baerts W, Van Bel F (2015). Brain-sparing in intrauterine growth restriction: Considerations for the neonatologist. Neonatology.

[63] Wishart DS (2007). HMDB: The human metabolome database. Nucleic Acids Res..

[64] Foucher, C. & Tubben, R. Lactic Acidosis. in *StatPearls [Internet]* 1–6 (StatPearls Publishing).

[65] Cui Z (2016). Improvement and evaluation of a staining method for measuring sperm lactate dehydrogenase C4 activity. Clin. Lab..

[66] Antiabong JF, Ball AS, Brown MH (2015). The effects of iron limitation and cell density on prokaryotic metabolism and gene expression: Excerpts from Fusobacterium necrophorum strain 774 (sheep isolate). Gene.

[67] Cardellini M (2018). 2-hydroxycaproate predicts cardiovascular mortality in patients with atherosclerotic disease. Atherosclerosis.

[68] NIH. Aminoacylase 1 deficiency. *Genetic and Rare Diseases Information Center* https://rarediseases.info.nih.gov/diseases/9741/aminoacylase-1-deficiency.

[69] Gioran, A. *et al.* Multi-omics identify xanthine as a pro-survival metabolite for nematodes with mitochondrial dysfunction. *EMBO J.* **38**, e99558 (2019). 10.15252/embj.201899558 PMC6418696 30796049

[70] Frolkis A (2010). SMPDB: The small molecule pathway database. Nucleic Acids Res..

[71] Molybdenum cofactor deficiency. *MedlinePlus [Internet], National Library of Medicine (US)* https://medlineplus.gov/genetics/condition/molybdenum-cofactor-deficiency/# (2020).

[72] Mendel, R. R. & Kruse, T. Cell biology of molybdenum in plants and humans. *Biochim. Biophys. Acta - Mol. Cell Res.* **1823**, 1568–1579 (2012). 10.1016/j.bbamcr.2012.02.007 22370186

[73] Garcia-Gil, M. *et al.* Emerging role of purine metabolizing enzymes in brain function and tumors. *Int. J. Mol. Sci.* **19** (2018). 10.3390/ijms19113598 PMC6274932 30441833

[74] Taibi G, Gaudio FD, Nicotra CMA (2008). Xanthine dehydrogenase processes retinol to retinoic acid in human mammary epithelial cells. J. Enzyme Inhib. Med. Chem..

[75] Taibi G, Paganini A, Gueli MC, Ampola F, Nicotra CM (2001). Xanthine oxidase catalyzes the synthesis of retinoic acid. J. Enzyme Inhib..

[76] Cui Y, Freedman JH (2009). Cadmium induces retinoic acid signaling by regulating retinoic acid metabolic gene expression. J. Biol. Chem..

[77] Guintivano, J., Aryee, M. J. & Kaminsky, Z. A. A cell epigenotype specific model for the correction of brain cellular heterogeneity bias and its application to age, brain region and major depression. *Epigenetics* **8**, 290–302. 10.4161/epi.23924 PMC3669121 23426267

[78] Leek JT, Storey JD (2007). Capturing heterogeneity in gene expression studies by surrogate variable analysis. PLOS Genet..

[79] Gagnon-Bartsch JA, Speed TP (2012). Using control genes to correct for unwanted variation in microarray data. Biostatistics.

[80] Gillespie MA (2020). Absolute quantification of transcription factors reveals principles of gene regulation in erythropoiesis. Mol. Cell.

[81] Hicks KD (2016). Interaction of bisphenol A (BPA) and soy phytoestrogens on sexually dimorphic sociosexual behaviors in male and female rats. Horm. Behav..

[82] Sullivan, A. W. *et al.* A novel model for neuroendocrine toxicology: Neurobehavioral effects of BPA exposure in a prosocial species, the prairie vole (Microtus ochrogaster). *Endocrinology* **155**, 3867–3881. 10.1210/en.2014-1379 PMC6285157 25051448

[83] Dobin A (2013). STAR: Ultrafast universal RNA-seq aligner. Bioinformatics.

[84] Anders S, Pyl PT, Huber W (2015). HTSeq—A Python framework to work with high-throughput sequencing data. Bioinformatics.

[85] R: A language and environment for statistical computing. *R Foundation for Statistical Computing* www.R-project.org (2017).

[86] Love MI, Huber W, Anders S (2014). Moderated estimation of fold change and dispersion for RNA-seq data with DESeq2. Genome Biol..

[87] Benjamini Y, Hochberg Y (1995). Controlling the false discovery rate: A practical and powerful approach to multiple testing. J. R. Stat. Soc. Ser. B.

[88] Santos, J. H., Mandavilli, B. S. & Van Houten, B. Measuring Oxidative mtDNA Damage and Repair Using Quantitative PCR. In *Mitochondrial DNA: Methods and Protocols* (ed. Copeland, W. C.) 159–176 (Humana Press, 2002). 10.1385/1-59259-284-8:159. 10.1385/1-59259-284-8:159 12013794

[89] Quiros PM, Goyal A, Jha P, Auwerx J (2017). Analysis of mtDNA/nDNA Ratio in Mice. Curr. Protoc. Mouse Biol..

[90] Bustin SA (2009). The MIQE guidelines: Minimum information for publication of quantitative real-time PCR experiments. Clin. Chem..

[91] Manza LL, Stamer SL, Ham A-JL, Codreanu SG, Liebler DC (2005). Sample preparation and digestion for proteomic analyses using spin filters. Proteomics.

[92] Wisniewski JR, Zougman A, Nagaraj N, Mann M (2009). Universal sample preparation method for proteome analysis. Nat. Methods.

[93] Chen EY (2013). Enrichr: interactive and collaborative HTML5 gene list enrichment analysis tool. BMC Bioinform..

[94] Kuleshov MV (2016). Enrichr: A comprehensive gene set enrichment analysis web server 2016 update. Nucleic Acids Res..

[95] Chong J (2018). MetaboAnalyst 4.0: Towards more transparent and integrative metabolomics analysis. Nucleic Acids Res..

[96] Jones JW, Pierzchalski K, Yu J, Kane MA (2015). Use of fast HPLC multiple reaction monitoring cubed for endogenous retinoic acid quantification in complex matrices. Anal. Chem..

[97] Kane MA, Folias AE, Napoli JL (2008). HPLC/UV quantitation of retinal, retinol, and retinyl esters in serum and tissues. Anal. Biochem..

[98] Kane MA, Napoli JL (2010). Quantification of endogenous retinoids. Methods Mol. Biol..

