# Supplementary figures and images for: Transcriptomic, proteomic, and metabolomic analyses identify candidate pathways linking maternal cadmium exposure to altered neurodevelopment and behavior
Source: Sci Rep. 2021 Aug 11;11:16302. doi: 10.1038/s41598-021-95630-2 (PMC8357970; doi:10.1038/s41598-021-95630-2)

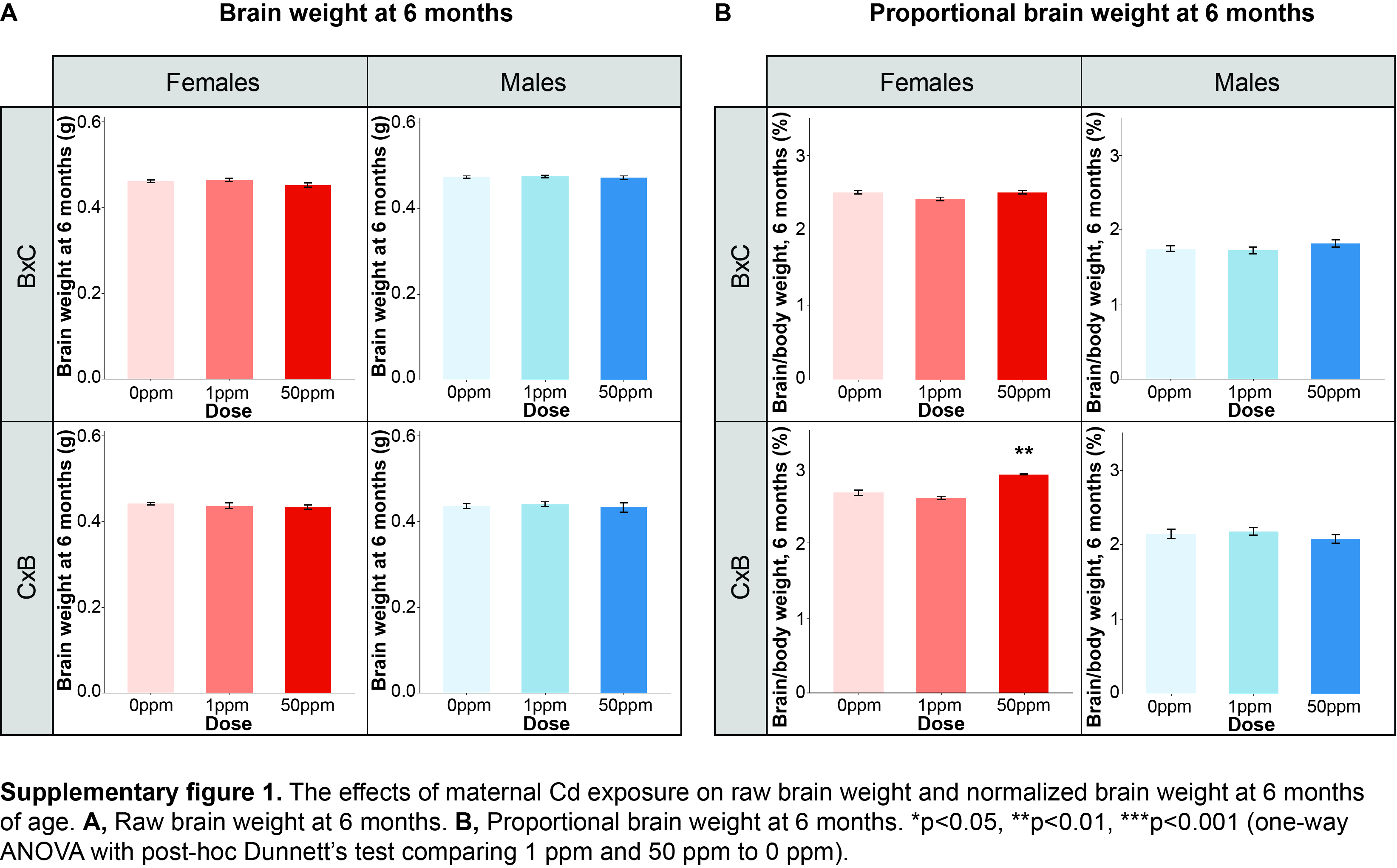

Supplement: Supplementary file 1 — Supplementary Information 1. [file 41598_2021_95630_MOESM1_ESM.jpg]

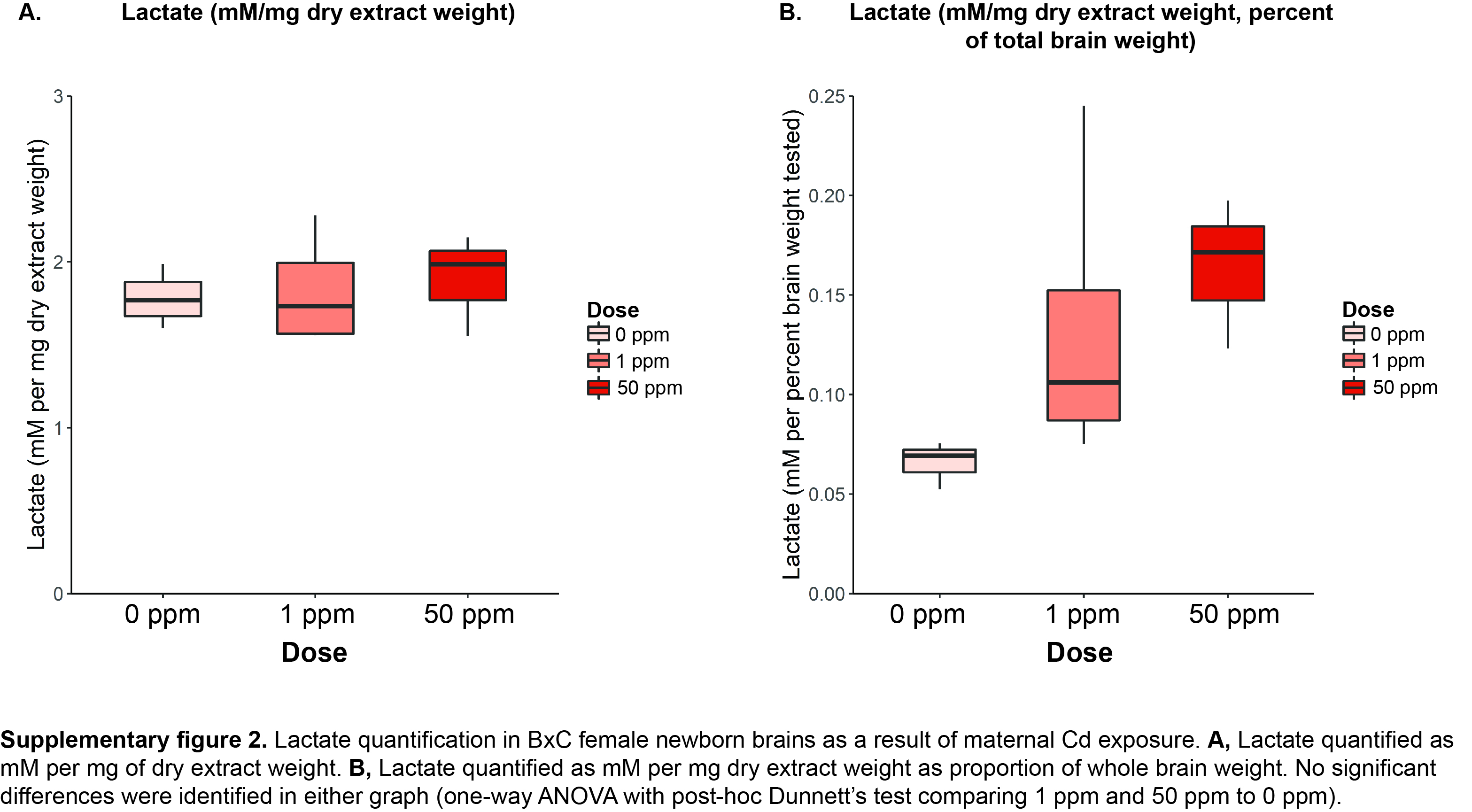

Supplement: Supplementary file 2 — Supplementary Information 2. [file 41598_2021_95630_MOESM2_ESM.jpg]
