# Supplementary material for: Transcriptomic, proteomic, and metabolomic analyses identify candidate pathways linking maternal cadmium exposure to altered neurodevelopment and behavior
Source: Sci Rep. 2021 Aug 11;11:16302. doi: 10.1038/s41598-021-95630-2 (PMC8357970; doi:10.1038/s41598-021-95630-2)
Supplement: Supplementary file 3 — Supplementary Information 3. [file 41598_2021_95630_MOESM3_ESM.docx]

Supplementary Material

Title Page

**Transcriptomic, proteomic, and metabolomic analyses identify candidate pathways linking maternal cadmium exposure to altered neurodevelopment and behavior**

Kathleen M Hudson^1,2^, Emily Shiver^1,2^, Jianshi Yu^3^, Sanya Mehta^1,2^, Dereje D Jima^2,4^, Maureen A Kane^3^, Heather B Patisaul^1,2^, Michael Cowley^1,2^*

^1^Department of Biological Sciences, North Carolina State University, Raleigh, NC, 27695, USA.

^2^Center for Human Health and the Environment, North Carolina State University, Raleigh, NC, 27695, USA.

^3^School of Pharmacy Mass Spectrometry Center, Department of Pharmaceutical Sciences, University of Maryland, Baltimore, MD 21201, USA.

^4^Bioinformatics Research Center, North Carolina State University, Raleigh, NC, 27695, USA.

*Author for correspondence: [macowley@ncsu.edu](mailto:macowley@ncsu.edu), 919-513-0818

Supplementary Figure 1 The effects of maternal Cd exposure on raw brain weight

and normalized brain weight at 6 months of age

Supplementary Figure 2 Lactate quantification in BxC female newborn brains as a

result of maternal Cd exposure

Supplementary Table 1 Behavior data

Supplementary Table 2 Newborn brain RNA-seq data aligned to B and C genomes

Supplementary Table 3 Proteomics raw data

Supplementary Table 4 Proteomics enrichment analysis

Supplementary Table 5 Metabolomics

Supplementary Table 6 Primer list

Supplementary Table 7 MIQE checklist

Supplementary Table 8 Statistics and source data
